# Unveiling the contribution of tumor-associated macrophages in driving epithelial-mesenchymal transition: a review of mechanisms and therapeutic Strategies

**DOI:** 10.3389/fphar.2024.1404687

**Published:** 2024-09-02

**Authors:** Yijia Zhang, Xiaofei Ding, Xue Zhang, Ye Li, Rui Xu, Hai-Jun Li, Daiying Zuo, Guang Chen

**Affiliations:** ^1^ Department of Pharmacy, Taizhou Second People’s Hospital (Mental Health Center affiliated to Taizhou University School of Medicine), Taizhou University, Taizhou, Zhejiang, China; ^2^ Department of Pharmacology, Shenyang Pharmaceutical University, Shenyang, China; ^3^ Department of Pharmacology, Taizhou University, Taizhou, Zhejiang, China

**Keywords:** TAM, tumor associated macrophage, TME, tumor microenvironment, EMT, epithelialmesenchymal transition, ECM, extracellular matrix, CSF1R, colony-stimulating factor 1 receptor

## Abstract

Tumor-associated macrophages (TAMs), fundamental constituents of the tumor microenvironment (TME), significantly influence cancer development, primarily by promoting epithelial-mesenchymal transition (EMT). EMT endows cancer cells with increased motility, invasiveness, and resistance to therapies, marking a pivotal juncture in cancer progression. The review begins with a detailed exposition on the origins of TAMs and their functional heterogeneity, providing a foundational understanding of TAM characteristics. Next, it delves into the specific molecular mechanisms through which TAMs induce EMT, including cytokines, chemokines and stromal cross-talking. Following this, the review explores TAM-induced EMT features in select cancer types with notable EMT characteristics, highlighting recent insights and the impact of TAMs on cancer progression. Finally, the review concludes with a discussion of potential therapeutic targets and strategies aimed at mitigating TAM infiltration and disrupting the EMT signaling network, thereby underscoring the potential of emerging treatments to combat TAM-mediated EMT in cancer. This comprehensive analysis reaffirms the necessity for continued exploration into TAMs’ regulatory roles within cancer biology to refine therapeutic approaches and improve patient outcomes.

## 1 TAMs at the crossroads of cancer: a dual-edged sword within the TME

Cancer, a multifaceted pathological entity, is increasingly understood through its interactions within the tumor microenvironment (TME), a diverse ecosystem comprising stromal cells, immune cells, and the extracellular matrix (ECM) (Hawkins et al., 2016). Among the immune cells, tumor-associated macrophages (TAMs) are of particular interest due to their significant, yet paradoxical roles in cancer progression (Iriondo et al., 2018).

TAMs represent a unique subset of myeloid cells within the TME, known to be associated with adverse clinical outcomes in a variety of cancers such as breast, lung, and pancreatic cancers (Akimoto et al., 2016). Their functionalities extend from phagocytosis and cytokine secretion to the remodeling of the ECM (Kitamura et al., 2015; Colwell et al., 2017). A significant aspect of TAMs’ influence is their facilitation of epithelial-mesenchymal transition (EMT) (Tekpli et al., 2019), a process enabling epithelial cells to acquire mesenchymal traits, thereby enhancing their migratory and invasive capacities (Oh et al., 2018). This phenotypic shift, marked by the downregulation of epithelial markers such as E-cadherin and the upregulation of mesenchymal markers including N-cadherin, Vimentin, and Fibronectin, is instrumental in the metastatic cascade. It enables malignant cells to detach from the primary tumor, intravasate into the bloodstream or lymphatic system, and subsequently establish secondary tumors in distant organs (Urbas et al., 2016).

Emerging research delineates the multifaceted roles of TAMs in orchestrating the EMT. TAMs exert their influence through the secretion of an array of bioactive molecules, including cytokines such as IL-6 and TGF-β, and various growth factors that synergistically activate EMT pathways in cancer cells (Kartikasari et al., 2021). This complex cocktail not only modulates gene expression to favor a mesenchymal phenotype but also disrupts cell-cell adhesion, enhancing migratory and invasive properties of cancer cells (Huang et al., 2020; Zhang et al., 2021).

TAMs play a pivotal role in orchestrating the TME, extending beyond their secretory roles to foster a pro-metastatic niche in collaboration with cancer-associated fibroblasts (CAFs) and other stromal cells. In this complex interplay, TAMs and CAFs collectively contribute to the creation of a pro-inflammatory milieu, characterized by the release of inflammatory mediators that sustain chronic inflammation and enhance mesenchymal phenotype (Habanjar et al., 2023; Nakamura et al., 2022). Moreover, TAMs express matrix metalloproteinases (MMPs) like MMP-9 (Niland et al., 2021), while CAFs contribute to ECM deposition through the production of fibronectin and collagen (Belhabib et al., 2021), leading to increased tissue stiffness and the creation of conduits that facilitate cancer cell invasion. The interaction between TAMs and CAFs in ECM remodeling is further facilitated by integrins and discoidin domain receptors (DDRs) on their surfaces, which mediate cell-ECM interactions and signal transduction involved in cancer cell migration and invasion (Lee et al., 2021; Gunaydin, 2021a).

Understanding the intricate relationship between TAMs and EMT is paramount for the development of innovative therapeutic strategies. This review commences with a detailed examination of the diversity and plasticity of macrophages, highlighting their aberrant functions within the TME. We then dissect the current knowledge on the role of TAMs in EMT induction and subsequent cancer cell metastasis. Given the heterogeneity of both tumors and TAMs, we underscore the variable impact of TAM-mediated EMT four cancer types. This review endeavors to elucidate the integral role of TAMs in cancer progression within the TME that could potentially impact patient outcomes.

## 2 Deciphering the complex nature of TAMs: Origins, classification, and the role in cancer dynamics

Macrophages are pivotal in tumor immune surveillance, capable of recognizing and eradicating malignant cells. However, in the context of cancer, these cells often undergo functional reprogramming, leading to the emergence of TAMs that can paradoxically support tumor growth and metastasis (Ratnam et al., 2019).

### 2.1 Lineage tracing and phenotype of TAMs

Within the TME, TAMs exemplify cellular adaptability, performing roles that transition from anti-tumorigenic to pro-tumorigenic in response to TME cues. TAM ontogeny is predominantly attributed to two lineages: the MerTK + CX3CR1+ resident tissue macrophages (RTMs) and the Ly6C + CCR2+ bone marrow-derived macrophages (BMDMs). The expression and kinase activity of MerTK are crucial for efferocytosis in human RTMs (Wanke et al., 2021), while CX3CR1 originates from embryonic CX3CR1+ precursors that migrate into tissues during embryogenesis and differentiate into various RTMs (Ensan et al., 2016). Ly6C is a classical surface marker highly expressed on inflammatory monocytes, facilitating their migration from the bone marrow to peripheral tissues (Miyake et al., 2024). CCR2, a chemokine receptor, is essential for guiding these monocytes during their transit to sites of inflammation, where they then differentiate into macrophages and partake in the immune response (Chen et al., 2023a).

RTMs, the custodians of tissue homeostasis, are established during embryogenesis, deriving from yolk sac and fetal liver progenitors. These cells disperse to various organs, where they acquire specialized functions unique to each tissue (Wu and Hirschi, 2019). For example, embryonically derived resident CD163+ Tim4+ omental macrophages have been shown to facilitate the metastatic progression of ovarian cancer (Etzerodt et al., 2020). RTMs are initially found in close proximity to tumor cells, where they contribute to the process of EMT and enhance tumor invasiveness. With tumor progression, however, RTMs are relegated to the periphery of the TME, making way for an influx of BMDMs that become predominant in the TME of both mouse models and human cases of NSCLC (Casanova-Acebes et al., 2021). Intriguingly, in breast cancer (BC), RTMs defy the anticipated dominance of BMDMs, playing an essential role in sculpting the tumor milieu (Hirano et al., 2023a). This finding underscores the importance of considering the origin of TAMs when studying their roles in tumor biology.

Conversely, BMDMs originate from a lineage of CD34^+^CD38^−^CD90^+^ hematopoietic stem cells within the bone marrow. These cells are lured into the tumultuous environment of the TME by a myriad of chemokines and growth factors. Monocytes characterized by Ly6C + CCR2+ surface markers can evolve into CD68^+^ macrophages, influenced by local mediators such as macrophage colony-stimulating factor (M-CSF) and granulocyte-macrophage colony-stimulating factor (GM-CSF) (Wu et al., 2020). The recruitment and functional roles of BMDMs are highly dynamic, often initiated by inflammatory signals, making them integral to the TME’s immunological landscape. Their propensity for rapid infiltration and response to inflammatory stimuli renders BMDMs pivotal in the later stages of tumor evolution and metastatic dissemination (Kielbassa et al., 2022). In melanoma tumors, F4/80+ TAM subsets have been primarily traced back to bone marrow progenitors, challenging the conventional belief of their derivation from skin-resident macrophages (Pizzurro et al., 2023). The findings accentuate the contributions of both RTMs and BMDMs to the intricacies of the TME. The distinct origins and developmental trajectories of these macrophages engender a diversity of roles within the TME, underscored by their context-dependent functions in cancer progression.

The phenotypic spectrum of TAMs is a complex mosaic, intricately crafted from the TME diverse array of cytokines, chemokines, growth factors, and metabolic by-products. This spectrum transcends the traditional binary categorization into CD80^+^CD86^+^ M1-like (anti-tumorigenic) and CD163+CD206+ M2-like (pro-tumorigenic) phenotypes, revealing a much broader continuum of TAM states (Fernandez et al., 2017; Pe et al., 2022). This continuum reflects the dynamic nature of the TME, with TAMs adopting hybrid phenotypes (Boutilier and Elsawa, 2021).

The advent of single-cell analytic technologies has marked a new epoch in TAM characterization, enabling a more nuanced understanding of their roles within the TME. For instance, in BC, such analyses have distinguished TAM subpopulations like TREM2+ TAMs, characterized by the expression of TREM2 and SPP1, and FOLR2+ TAMs, identifiable by FOLR2 and CD206 markers. These refined subdivisions underscore the potential for developing targeted therapies aimed at modulating TAM-driven oncogenic processes, opening up new avenues for cancer treatment (Nalio Ramos et al., 2022).

Furthermore, the characterization of TAMs varies across different models and tumor types, reflecting the adaptability and diversity of these cells in response to unique TME. For instance, a study utilizing a larval *Drosophila* model, which harbors a dominant-active version of the Ras oncogene (RasV12), shed light on dysplastic growth during early tumor progression. Analysis of hemocytes in this model revealed five distinct clusters, each exhibiting unique gene expression profiles. This suggests that, even in *Drosophila*, TAMs can be categorized into multiple subtypes based on their molecular signatures, hinting at the complex roles these cells play in tumor biology (Khalili et al., 2023).

In human glioblastoma, research has unveiled a broad spectrum of TAM subtypes that extends well beyond the conventional M1/M2 dichotomy. This includes as many as 11 distinct subtypes, encompassing hypoxic, proliferating, and chemokine-producing TAMs, among others. Such findings underscore the heterogeneity and complexity of TAM populations within tumors, challenging the traditional binary classification and highlighting the nuanced roles these cells play in tumorigenesis (Anand et al., 2022).

### 2.2 Heterogeneity and plasticity of TAMs

The remarkable heterogeneity and plasticity of TAMs within the TME are shaped by a complex web of signals, steering them towards roles that either support or counteract tumor growth. M1 polarization is incited by pro-inflammatory mediators such as interferon gamma (IFN-γ), intratumoral bacterial lipopolysaccharide (LPS), and tumor necrosis factor alpha (TNF-α), orchestrating the activation of transcription factors including STAT1, interferon regulatory factor 5 (IRF5), and NF-κB (Chin et al., 2021). Conversely, M2 polarization is fostered by anti-inflammatory cues, notably IL-4, IL-13, and IL-10, which mobilize transcription factors like STAT6, IRF4, and PPAR-γ (Ren et al., 2021).

The conventional dichotomy of TAMs into M1 and M2 macrophages has undergone significant scrutiny with the emergence of scRNA-seq technologies. These analyses have revealed that TAMs often exhibit a blend of M1 and M2 characteristics at the single-cell level, demonstrating a more intricate spectrum of TAM phenotypes within the TME. This methodological advancement has also enabled the identification of multiple TAM subsets across different cancer types, each characterized by distinct gene expression profiles and functional roles. For instance, in pancreatic ductal adenocarcinoma (PDAC), scRNA-seq has delineated TAMs into seven distinct clusters, with the CCL2+CCL3 + cluster1 and TOP2A + cluster3 being particularly relevant to PDAC pathology. These findings highlight the exceptional diversity and context-specific nature of TAMs in various tumor settings, prompting a reconsideration of the simplistic M1/M2 paradigm and encouraging a deeper exploration of TAM functions in cancer (Yang et al., 2023a).

In addition to cytokine engagement, the metabolic landscape of the TME plays a critical role in modulating TAM polarization, with factors like lactate accumulation and the presence of lipid-rich apoptotic debris through efferocytosis significantly influencing TAM behavior, potentially even surpassing the impact of canonical cytokine signaling. The TME’s enriched metabolic milieu, characterized by these components, profoundly dictates TAM functionality (Zhang et al., 2022; Cheng et al., 2020; Okikawa et al., 2022; Kloosterman and Akkari, 2023). The accumulation of lipid droplets, in particular, has been shown to drive TAMs towards an M2-like phenotype, conducive to tumor progression. This process involves the hydrolysis of stored triglycerides into free fatty acids (FFAs) by enzymes such as adipose triglyceride lipase (ATGL) and hormone-sensitive lipase (HSL), underscoring the link between lipid metabolism and TAM-mediated tumor support mechanisms (Wu et al., 2019). TAMs exhibit remarkable metabolic flexibility, adapting their metabolic pathways to the diverse and fluctuating demands of the TME. This adaptation enables TAMs to efficiently utilize glucose, amino acids, and lipids, tailoring their energy production and biosynthetic activities to optimally support tumor growth and survival (Xiao et al., 2023).

Epigenetic mechanisms further refine the functional spectrum of TAMs. In M2-like TAMs, DNA methyltransferase 3B (DNMT3B) modulates the arginase 1 (ARG1) gene, integral to the M2 phenotype, where DNMT3B deficiency augments ARG1 expression in BMDMs (Qin et al., 2022). Similarly, the inhibition of histone deacetylase 6 (HDAC6) has been shown to reduce M2 polarization, emphasizing the role of histone modifications in determining TAM functionality (Shi et al., 2022). The overexpression of HDAC2 in M2-like TAMs has been associated with adverse outcomes for lung cancer patients. Targeting the HDAC2-SP1 axis may disrupt the pro-tumorigenic activities of M2-like TAMs, thereby impairing their support for tumor progression (Zheng et al., 2023).

The metabolic dichotomy between M1 and M2 macrophages is underscored by their respective reliance on glycolysis showing interruptions in the tricarboxylic acid TCA cycle, which leads to the stabilization of hypoxia-inducible factor 1-alpha (HIF1-α) and fatty acid synthesis for pro-inflammatory activities (Qian et al., 2024), and oxidative phosphorylation (OXPHOS) alongside fatty acid oxidation for anti-inflammatory and tissue repair functions, but the glycometabolism of TAMs within the TME becomes more complex and diverse (Zhang et al., 2022; Kes et al., 2020), reflecting their adaptation to the diverse conditions of the TME (Wculek et al., 2022; Elia and Haigis, 2021).

TAMs display remarkable metabolic flexibility, oscillating between glycolysis and OXPHOS in response to the dynamic oxygen and nutrient availability within the TME. This metabolic plasticity is highlighted by the altered expression of key metabolic components in TAMs, including an upsurge in glucose transporters like GLUT1 and glycolytic enzymes such as hexokinase 2, signifying a propensity towards aerobic glycolysis (Shin and Koo, 2021). Furthermore, modifications in lipid metabolism are apparent through the enhanced expression of enzymes involved in fatty acid synthesis, like fatty acid synthase, alongside shifts in cholesterol metabolism, which may contribute to the production of pro-inflammatory and immunosuppressive lipid mediator (Li et al., 2022).

**FIGURE 1:**
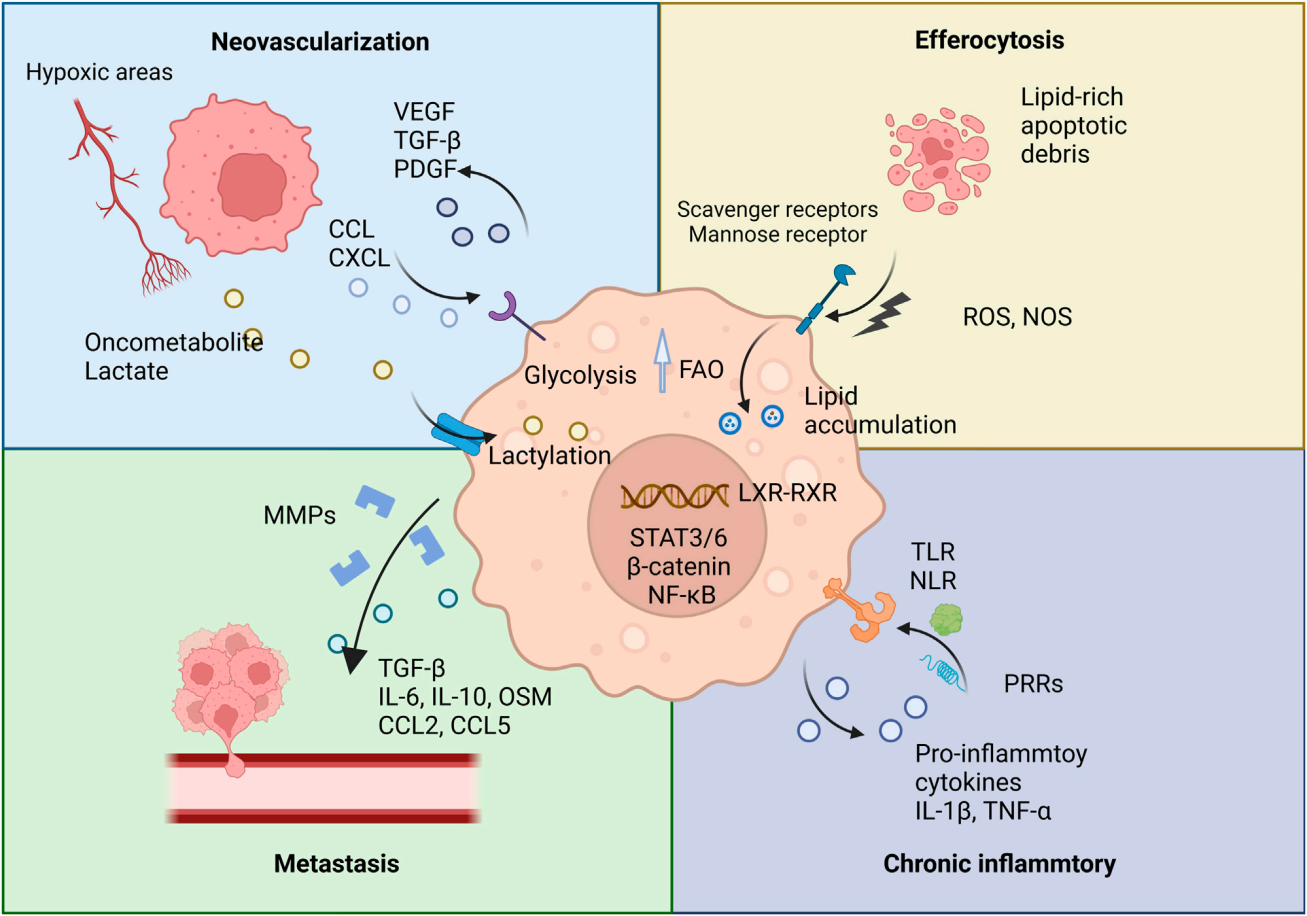
TAMs and their pro-tumorigenic functions. This figure illustrates the various pro-tumorigenic roles of TAMs within the TME. TAMs contribute to several key processes that promote tumor progression.

The process of efferocytosis, the engulfment of apoptotic cells, further influences TAM metabolism by inducing fatty acid oxidation, which not only supports TAM survival in the nutrient-depleted TME but may also enhance the stability of the TME (Kloosterman and Akkari, 2023). Consequently, targeting TAM lipid metabolism presents a promising strategy in cancer therapy. For example, arenobufagin has been shown to affect macrophage polarization by modulating PCSK9-mediated cholesterol metabolism. By inhibiting the PCSK9/LDL-R pathway, arenobufagin promotes M1-type polarization, illustrating the therapeutic potential of manipulating TAM metabolic pathways (Figure 1) (Li et al., 2024).

### 2.3 Dual role of TAMs in tumor progression and suppression

The dual role of TAMs is highlighted by their controversial relationship with cancer prognosis. Some studies associate TAMs with poor prognosis, while others report better outcomes in different cancer types (Liu et al., 2021).

Theoretically, the TME significantly influences TAMs, often impairing their antigen-presenting capabilities and steering them towards an M2-like phenotype, linked with tumor-promoting activities such as immunosuppression, angiogenesis, and tissue remodeling (Cheng et al., 2020; Wischhusen et al., 2020). Yet, the adaptability and diversity of TAMs are remarkable, with their functions being profoundly context-specific. While M1-like TAMs are generally associated with anti-tumorigenic properties, certain TME conditions can paradoxically lead them to contribute to tumor dissemination (You et al., 2022). In lung cancer, CXCL9+ M1 TAMs recruit tissue-resident T cells and facilitate fatty acid uptake, enhancing their metabolic fitness and anti-tumor effects (Eva et al., 2020).

The activation of TAMs using toll-like receptor ligands, for example, can induce a transition towards a pro-inflammatory phenotype. This phenotypic shift not only elevates their antigen-presentation capabilities but also amplifies cytokine secretion, ultimately bolstering the activation of antitumor T cells (Zhang et al., 2021b). TAMs also facilitate antibody-dependent cellular cytotoxicity (ADCC) and phagocytosis, contributing to vascular disruption and tumor necrosis, thereby destabilizing the TME and impeding cancer progression (Wettersten et al., 2019; Mantovani et al., 2017). Current research explores therapeutic targeting of macrophages, including chimeric antigen receptor macrophages (CAR-M), highlighting their potential in cancer treatment (Klichinsky et al., 2020).

## 3 The role of TAMs in facilitating EMT during cancer progression

TAMs are central to the orchestration of EMT, a crucial process in cancer metastasis. Through the secretion of signaling molecules, direct interactions with cancer cells, and modulation of immune responses, TAMs conduct the complex orchestra that regulates EMT. Understanding this interplay between TAMs and cancer cells not only elucidates the mechanisms of EMT but also reveals potential therapeutic targets.

### 3.1 TAM-secreted signaling molecules: Key conductors in the EMT orchestra

TAMs significantly influence the TME by secreting a diverse array of signaling molecules that drive the EMT process. This section explores the interactions between TAM-derived factors, such as growth factors, cytokines, and chemokines—including TGF-β, IL-6, and oncostatin M (OSM)—and their roles in inducing EMT.

#### 3.1.1 TGF-β signaling

TGF-β, abundantly secreted by TAMs, emerges as a pivotal orchestrator of the EMT. The multifaceted roles of TGF-β in EMT modulation, highlighting its ability to actuate downstream transcriptional effectors, particularly Smad2/3, thereby influencing the expression of EMT-associated transcription factors (EMT-TFs) (Cai et al., 2019).

##### 3.1.1.1 Direct promotion of EMT

In lung squamous cell carcinoma, increased secretion of TGF-β1 from TAMs has been confirmed and promotes EMT by the role of TGF-β in the Smad/zinc finger e-box binding homeobox (ZEB) pathway (Sumitomo et al., 2023). Addressing this mechanism, innovative approaches like macrophage membrane-coated nanoparticles loaded with SD-208 (Mφ-SDNP), a TGF-βR1 kinase inhibitor, aim to directly inhibit the TGF-β signaling pathway, thereby preventing the initiation of EMT in cancer cells. SD-208 within the Mφ-SDNP inhibits the TGF-β receptor’s kinase activity, blocking the downstream signaling pathways that lead to EMT. In addition to targeting cancer cells, Mφ-SDNP also prevents the polarization of macrophages to the M2 type. Treatment with Mφ-SDNP results in a significant decrease in mRNA and protein levels of EMT markers. Furthermore, it increases the population of cytotoxic T lymphocytes within the tumor, thereby enhancing the effectiveness of immune checkpoint inhibitors (Kim et al., 2023).

In colorectal cancer (CRC), TAM-derived TGF-β is known to escalate EMT by enhancing phosphorylated SMAD2/3 and SMAD4 levels, exemplifying the crucial role of TAMs in this regulatory cascade (Cai et al., 2019). Notably, it also has been implicated in augmenting HIF-1α levels, leading to the upregulation of tribbles pseudokinase 3 (TRIB3), which in turn activates the Wingless/Integrated (Wnt)/β-catenin pathway, thus propelling EMT and lung metastasis (Liu C. et al., 2021). Besides, collagen triple helix repeat containing 1 (CTHRC1), a protein highly expressed in CRC, can upregulate chemokine (C-C motif) ligand 15 (CCL15) via the TGF-β/Smad signaling pathway, enhancing TAM infiltration and creating a feedback loop that further propels the disease progression (Liu et al., 2024). In cholangiocarcinoma, TAM-secreted TGF-β has been shown to activate Gli2, intensifying EMT processes and maintaining endoplasmic reticulum (ER) homeostasis (Chen Z. et al., 2023). The anticancer agent emodin demonstrates potential in curtailing TGF-β secretion by TAMs, consequently mitigating TAM-induced EMT and cellular stemness (Liu et al., 2020a).

##### 3.1.1.2 Chemotherapy resistance

TAM-secreted TGF-β fosters chemotherapy resistance, with prolonged exposure linked to elevated EMT, stemness, and drug resistance (Katsuno et al., 2019). For instance, in esophageal squamous cell carcinoma (ESCC), TAM-derived TGF-β has been associated with cisplatin resistance through the enhancement of cancer stem cell (CSC) properties (Yang et al., 2023b). Conversely, there is evidence that certain pharmaceutical agents can modulate TAM behavior to counteract these effects. Simvastatin, a cardiovascular drug, shows promise in re-polarizing TAMs towards an anti-tumorigenic phenotype and reducing TGF-β secretion, decreasing EMT and increasing lung cancer cell sensitivity to paclitaxel (Jin et al., 2019).

##### 3.1.1.3 Feedback loop and regulation mechanisms

TAMs reinforce TGF-β feedback loops within cancer and stromal cells, amplifying EMT and cancer progression. For instance, the E3 ubiquitin ligase RAD18 has been shown to strengthen a positive feedback loop between triple-negative breast cancer (TNBC) cells and macrophages mediated by TGF-β, thereby increasing cellular stemness (Yan et al., 2022). TGF-β also prompts fibroblast growth factor 2 (FGF2) secretion in CAFs, which subsequently influences TAM polarization in gastric cancer (GC), significantly propelling migration, invasion and CSC traits (Li et al., 2023a). In bladder cancer, cancer cells prompt TAMs to secrete TGF-β, facilitated by the HIF-1α signaling pathway—a response linked to aerobic glycolysis within the tumor milieu. In a reciprocal interaction, TGF-β released by TAMs fosters glycolysis in bladder cancer cells via the Smad2/3 signaling pathways. This interaction not only amplifies the CSCs but also promotes EMT process (Shen et al., 2023).

##### 3.1.1.4 Receptor modulation

Additionally, TAMs alter the ratio of TGF-β receptors on neoplastic cells, modulating EMT induction. In PDAC, for instance, TAMs have been observed to downregulate the expression of TGF-β receptor 3 (TGFBR3) while concurrently upregulating TGFBR1 and TGFBR2. This modulation in receptor expression enhances the sensitivity of PDAC cells to TGF-β, thereby facilitating EMT through increased SMAD3 signaling and the subsequent upregulation of EMT-TFs (Yin et al., 2019).

##### 3.1.1.5 ECM stiffening

In BC, macrophage-derived TGF-β is a key player in stimulating the transdifferentiation of fibroblasts and inducing the expression of collagen crosslinking enzymes such as lysyl oxidase (LOX) and lysyl hydroxylase 2 (LH2). The activity of these enzymes not only increases the tensile strength and structural integrity of the collagen network but also contributes to the pathological stiffening of the tumor stroma. A positive correlation exists between the TAM marker CD163 and collagen crosslinking enzymes LOX and PLOD2, but not LOXL2, suggesting a regulatory relationship between TAMs and ECM stiffening. This relationship underscores the critical role of macrophage-secreted TGF-β in shaping the extracellular matrix and influencing tumor metastasis in BC (Maller et al., 2021).

##### 3.1.1.6 Stromal cell modulation

TAMs also augment TGF-β production and modulate the functions of other stromal cells to advance tumor progression. Studies have highlighted that TAMs secrete TGF-β1, which plays a pivotal role in the differentiation of CAFs from mesenchymal stromal cells (MSCs), as well as in enhancing their synthesis of ECM proteins. These CAFs, in turn, secrete TGF-β1, contributing to the induction of EMT and the development of drug resistance in neuroblastoma cells (Louault et al., 2022a). Targeting the TGF-β1/IL-6 pathway has been shown to reduce tumor burden and metastasis *in vivo* while enhancing neuroblastoma cell sensitivity to chemotherapy, underscoring the therapeutic potential of interrupting this pathway (Louault et al., 2022b).

#### 3.1.2 IL-6 family signaling

IL-6, secreted by TAMs, is recognized for its pro-inflammatory role within TME, particularly under hypoxic conditions and in response to inflammatory stimuli. The importance of IL-6 as a biomarker in various cancers is increasingly recognized, highlighting its role in oncogenesis and tumor progression (Zhang et al., 2020; Unver and McAllister, 2018), with TAM-derived IL-6 pivotal in activating the STAT3 cascade, thereby upregulating EMT-TFs and propelling tumor progression (Siersbaek et al., 2020; Huang et al., 2022).

##### 3.1.2.1 Mechanisms of IL-6 elevation

###### 3.1.2.1.1 YY1 transcriptional complex

For example, in M2-like macrophages, the yin yang 1 (YY1) transcriptional complex amplifies IL-6 expression through long-range chromatin interactions between an M2-specific IL-6 enhancer and the IL-6 promoter. Regulation of YY1 by the IL-4/STAT6 pathway, along with the phase separation of the YY1 complex during M2 macrophage polarization, are key mechanisms in the elevation of IL-6 expression (Chen et al., 2023c).

###### 3.1.2.1.2 Metabolic influence

In oral squamous cell carcinoma (OSCC), the glycolytic enzyme alpha-enolase (ENO1) is released into the TME, eliciting an IL-6 surge in TAMs that bolsters ENO1 expression and propels the EMT process (Lin Y. et al., 2023). Additionally, lactate, a prevalent metabolic byproduct in the TME, induces lactylation of retinoic acid receptor gamma (RARγ) in macrophages, leading to increased IL-6 secretion and activation of the oncogenic STAT3 signaling pathway (Li X.-M. et al., 2024).

##### 3.1.2.2 Pro-inflammatory role of IL-6

The IL-6 family, known for its pro-inflammatory properties, empowers M1 phenotype TAMs to drive tumor progression. In OSCC, M1-like TAMs are characterized by a significant surge in IL-6 secretion, which enhances EMT and fosters cellular stemness. This effect is mediated through the IL-6/STAT3/thrombospondin 1 (THBS1) signaling pathway, leading to an upregulation of MMP14 expression (You et al., 2022). Additionally, the upregulation of high mobility group box 1 (HMGB1) in OSCC has been observed to induce M1 polarization of macrophages. This polarization leads to an augmented secretion of IL-6, which furthers migration and invasion capabilities of OSCC cells via the activation of the NF-κB/IL-6 signaling axis, highlighting a critical mechanism through which pro-inflammatory M1 TAM signals contribute to cancer aggressiveness (Jiang et al., 2024).

##### 3.1.2.3 Feedback loop and regulation mechanisms

In CRC, exosomes from tumor cells, rich in circular RNAs, activate TAMs’ NF-κB pathway, leading to IL-6 secretion and triggering tumor cells’ Janus kinase 2 (Jak2)/STAT3 pathway. This enhances chemokine CCL2 levels, contributing to EMT and macrophage recruitment (Zhou et al., 2023). Likewise, TAMs conditioned by CRC notably elevate IL-6 secretion, activating the Jak2/STAT3 pathway and indirectly boosting forkhead box Q1 (FoxQ1) expression in cancer, thus reinforcing EMT and cancer stem cell attributes (Wei et al., 2019a). Intriguingly, in CRC, macrophages amplify IL-6 production from cancer cells themselves, suggesting that a substantial portion of IL-6 present in CRC’s TME might primarily be derived from the tumor cells rather than the macrophages (Gao et al., 2018). Besides, IL-6 is capable of inducing its own expression in both macrophages and gastric epithelial cells, creating an autocrine and paracrine positive feedback loop. This loop is further potentiated by *H. pylori* stimulation, which may initially induce an M1 polarization characterized by an inflammatory phenotype. However, chronic infection with *Helicobacter pylori* can lead to a shift towards M2 polarization, driven by continuous IL-6 production, highlighting the dynamic nature of TAM polarization and its impact on cancer progression (Yu et al., 2024).

##### 3.1.2.4 Other pathways

The DNMT family, especially DNMT1, influences TAM polarization, with TAM-induced IL-6/STAT3/ZEB1 signaling promoting DNMT1 expression in BC, correlated with elevated DNMT1, CD163, and ZEB1 expression in patients’ breast tissues (Li Z. et al., 2022). Besides, BC cells release exosomal circular RNA cSERPINE2, which is taken up by TAMs. This interaction leads to an increase in MALT1 (mucosa-associated lymphoid tissue lymphoma translocation protein 1) levels within TAMs, subsequently activating the NF-κB pathway. Activation of this pathway triggers the secretion of IL-6 by TAMs, which then enhances the levels of eukaryotic initiation factor 4A3 (EIF4A3) and the chemokine CCL2 in tumor cells. This cascade establishes a positive feedback loop that not only increases the biogenesis of cSERPINE2 within tumor cells but also promotes further recruitment of TAMs and facilitates the invasion capabilities of BC cells (Boxuan Zhou et al., 2023).

In lung cancer, IL-6 mediates interactions between cancer cells and microglia via Jak2/STAT3 signaling, aiding brain metastasis, a significant mortality driver. Brain-metastatic lung cancer cells secrete IL-6, prompting microglia’s M2 polarization, which facilitates metastatic colonization (Jin et al., 2022). TAMs secrete IL-6, which activates the JAK2/STAT3 pathway through autocrine secretion in TAMs. This activation leads to the stimulation of CCAAT/enhancer-binding protein β (C/EBPβ), which in turn promotes the transcription and further expression of IL-6, thus creating an IL-6-STAT3-C/EBPβ-IL-6 positive feedback loop. This loop is significant in perpetuating the inflammatory and tumor-promoting environment within the TME (Hu et al., 2024). Targeting IL-6 signaling, particularly through dual STAT3 and IL-6R inhibition, emerges as a viable strategy to restrict migration and metastasis (Hu et al., 2024; Méndez-Clemente et al., 2022).

##### 3.1.2.5 OSM-another key IL-6 family member in tumor progression

OSM, characterized by its pro-inflammatory properties, plays a pivotal role in the dynamics between TAMs and tumor cells (Masjedi et al., 2021). OSM is abundantly expressed in M2-polarized THP-1 derived macrophages, triggering the STAT3 signaling pathway to initiate EMT in BC (Guo et al., 2013). In glioblastoma, OSM released by TAMs is linked to the enhancement of mesenchymal-like (MES-like) cellular states, correlating with increased tumor aggression and poorer clinical outcomes (Weidner et al., 2023). In early-onset tongue cancer, areas that exhibit vascular mimicry show a high prevalence of macrophage markers, including OSM, suggesting its role in supporting tumor growth and enhancing metastatic potential (Marina et al., 2023).

In BC, Gr1+CD11b+ cells, educated by the tumor, secrete OSM and IL6, significantly expanding the metastatic SCA1+ cell population within the cancer (Peyvandi et al., 2024). Mechanistically, OSM has been shown to induce a mesenchymal phenotype in PDAC cells through the activation of the mitogen-activated protein kinase (MAPK) signaling pathway. This activation is pivotal for the stemness traits induced by OSM, as evidenced by comprehensive transcriptomic analyses. The perpetuation of this effect involves a feed-forward loop, where OSM stimulates transcriptional upregulation of the OSM receptor (OSMR), thus reinforcing the mesenchymal phenotype and stemness characteristics in PDAC cells (Polak et al., 2023).

**FIGURE 2:**
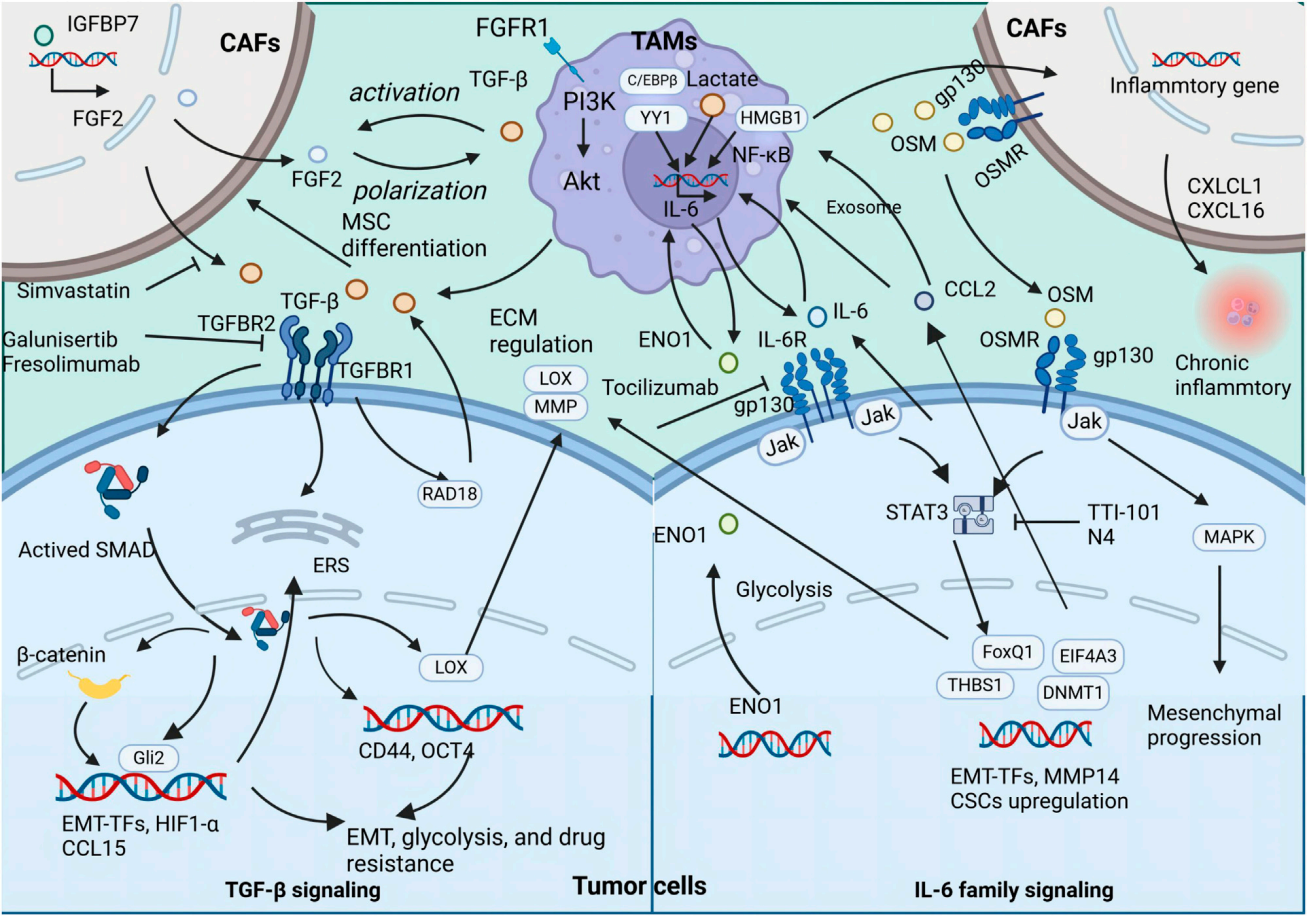
The secretion of TGFβ, IL-6, and OSM by TAMs exert both direct and indirect influences on the promotion of tumor metastasis and CSC properties The illustration depicts the signaling cascade of TAM-secreted TGFβ, IL-6, and OSM that promotes EMT and augments the stemness of neoplastic cells.

Recent studies have explored the interplay mediated by OSM between TAMs and other stromal cells, such as CAFs. OSM secreted by TAMs has been found to stimulate the expression of inflammatory genes in CAFs, supporting tumor survival and promoting metastasis. In PDAC mouse models, the absence of Osm significantly reduces the metastatic spread of the tumor, underlining the critical role of OSM in tumor progression (Lee et al., 2021). In BC, OSM produced by myeloid cells, including TAMs, activates OSMR on CAFs, inducing secretion of chemokines such as CXCL1 and CXCL16, which attract more TAMs to the tumor site, reinforcing a pro-inflammatory feedback loop that facilitates lung metastasis (Araujo et al., 2022). Recent reports highlight the significance of heterocellular OSM-OSMR signaling in promoting tumor metastasis through TAMs, tumor cells, and other stromal cells intercellular communication (Figure 2).

#### 3.1.3 Chemokine signaling

Chemokines derived from TAMs are increasingly recognized as critical facilitators of EMT in malignant cells, drawing significant attention for their potential as targets in novel therapeutic strategies aimed at halting tumor progression. These chemokines, emanating from diverse cellular subsets within the TME—including immune, endothelial, and stromal cells (Ozga et al., 2021) (Kumari and Choi, 2022).

##### 3.1.3.1 CCL18

###### 3.1.3.1.1 Receptor and mechanism

Phosphatidylinositol transfer protein, membrane-associated 3 (PITPNM3), has been identified as the receptor for CCL18, a chemokine linked with EMT induction. Subsequent investigations have unveiled the role of CCL18-PITPNM3 signaling in promoting extracellular matrix adherence and cellular migration in BC (Chen et al., 2011). Recent findings indicate that CCL18, upregulated in M2 macrophages, facilitates metastasis in squamous cell carcinoma of the head and neck (SCCHN) (She et al., 2018). The underlying mechanisms of CCL18-induced EMT in SCCHN involve activation of the metadherin (MTDH)/NF-κB cascade (Qin et al., 2019). Moreover, TAM-derived CCL18 can stimulate Annexin A2 (AnxA2), which then activates the EMT through the PI3K/Akt/GSK3β/Snail pathway in BC, and similar activation of PI3K/Akt in gallbladder cancer (GBC) promotes cell migration (Zhao et al., 2019; Zhou et al., 2018).

In ESCC, advanced analyses integrating single-cell transcriptomic sequencing with bulk microarray data have uncovered that CCL18, released by TAMs, drives tumor cell proliferation via the JAK2/STAT3 signaling pathway, correlating with a poorer prognosis in ESCC (Sui et al., 2023). Further investigations have identified the immune responsive gene 1 (IRG1) as a regulator of CCL18 secretion by TAMs. It has been found that the overexpression of IRG1 in macrophages leads to a reduction in CCL18 levels. This decrease in CCL18 secretion results in the suppression of malignant behaviors in intrahepatic cholangiocarcinoma (ICC) cells, suggesting a promising therapeutic avenue (Zhou et al., 2024).

###### 3.1.3.1.2 Feedback loop and regulation mechanisms

In HNSCC, TAMs expressing SPP1 give rise to a pro-angiogenic SPP1+CCL18+ TAM subset, implicated in tumor growth and metastasis. These SPP1+CCL18+ TAMs are associated with increased tumor growth and metastasis, primarily due to their high expression levels in metastasis-associated and EMT pathways (Wu et al., 2024). Furthermore, in the context of perineural invasion (PNI) in PDAC, elevated CCL18 levels in TAMs engage in paracrine interactions with Schwann cells, integral to the neural microenvironment, thereby facilitating PDAC progression (Zhang et al., 2024).

A notable feedback loop involving CCL18 in ovarian cancer has been shown to increase metastasis through a cascade beginning with CCL18-induced ZEB1 upregulation in cancer cells, which in turn leads to increased secretion of M-CSF. The heightened levels of M-CSF contribute to an enhanced TAM phenotype, fostering a TME conducive to cancer metastasis. The interplay among CCL18, ZEB1, and M-CSF accelerates the metastatic process (Long et al., 2021). CCL18 also mediates communication between TAMs and CAFs in BC, fostering chemoresistance. CCL18 promotes the transition of CAFs towards a CD10^+^ GPR77+ phenotype, a change associated with the acquisition of stemness properties and chemoresistance (Zeng et al., 2022).

##### 3.1.3.2 CCL2

The role of CCL2 varies depending on the tumor stage. It can promote or suppress tumors based on the immune cells it recruits, such as TAMs, NK cells, and T cells (Archer et al., 2023).

###### 3.1.3.2.1 Mesenchymal promotion mechanisms

In TNBC, CCL2 is known to activate Akt signaling, leading to β-catenin nuclear translocation and promoting both stemness and EMT through the CCL2/Akt/β-catenin pathway (Chen X. et al., 2022). The impact of this signaling pathway is further magnified by the direct interaction between β-catenin and the CCL2 promoter, which amplifies the characteristics of breast cancer stem cells, thus potentiating the aggressive nature of TNBC (Zhang F. et al., 2021; Tigue et al., 2023). Moreover, CCL2 secreted by TAMs has been linked to the induction of tamoxifen resistance in breast cancer cells through the PI3K/Akt/mammalian target of rapamycin (mTOR) pathway (Li et al., 2020).

In renal cell carcinoma, CCL2 secreted by M2 macrophages increases muscleblind like splicing regulator 2 (MBNL2) expression, which in turn stabilizes B-cell lymphoma 2 (Bcl-2) mRNA, leading to the inhibition of Beclin 1-dependent autophagy and endowing RCC cells with invasion properties (He et al., 2023).

###### 3.1.3.2.2 Feedback loop and regulation mechanisms

CCL2 also serves as a chemokine for TAMs, enhancing their pro-tumoral functions and infiltration. For instance, Wnt5a + TAMs activate the Wnt5a-CaMKⅡ-ERK cascade, increasing CCL2 production and enhancing TAMs and CRC cells migration *in vitro*. (Liu et al., 2020b). In NSCLC, a feedback loop involving circHSPB6, let-7a-2-3p, and CCL2 promotes TAM polarization to the M2 phenotype, supporting infiltration and fostering EMT (Li et al., 2023b). In ovarian cancer, MYBL2 activates CCL2 transcription, recruiting macrophages and promoting their M2 polarization (Pan et al., 2023).

###### 3.1.3.2.3 Therapeutic potential

Given these dynamics, combination therapy involving CCR2 antagonists and anti-PD-1 antibodies has shown greater efficacy in suppressing tumor growth and lung metastasis in solid cancer compared to control or monotherapy, highlighting the potential for targeted intervention in CCL2-mediated pathways to enhance cancer treatment outcomes (Weng et al., 2024; Lin et al., 2024).

##### 3.1.3.3 CCL22

###### 3.1.3.3.1 Mesenchymal promotion mechanisms

Recent studies have illuminated the role of CCL22, particularly its elevated expression in TAMs, in activating diverse signaling pathways that enhance the metastatic capabilities of tumor cells.

In ESCC, for instance, TAM-derived CCL22 can hyperactivate focal adhesion kinase (FAK) in ESCC cells by promoting the formation of a complex with diacylglycerol kinase alpha (DGKα). This activation leads to increased tumor cell migration and invasion, which can be mitigated by pharmacological inhibition of FAK or by employing anti-CCL22 treatments (Chen J. et al., 2022). Furthermore, CCL22 has been found to stimulate DGKα activity in tumor cells, suppressing NADPH oxidase 4 (NOX4) and averting cisplatin-induced ROS overproduction. This mechanism is closely tied to the development of chemoresistance in ESCC (Chen et al., 2024). Additionally, the activation of RSK4 in ESCC cells can elevate CCL22 expression, enhancing the release of soluble ICAM-1 and indirectly influencing STAT3 phosphorylation. Experiments with recombinant CCL22 and neutralizing antibodies have demonstrated significant alterations in the expression of EMT markers and tumor cell invasion (He et al., 2024). Moreover, TAM-secreted CCL22 can activate FAK, which subsequently mediates the phosphorylation of Gli1 at specific residues, boosting Gli1’s transcriptional activity and propelling ESCC progression. TAMs positive for CCL22 markedly enhance the invasive capacity and anchorage-independent growth of ESCC cells (Chen J. et al., 2023). Pexidartinib targets the M-CSFR to inhibit TAMs, leading to a reduction in CCL22 production. This mechanism underpins the rationale for clinical trials exploring the use of pexidartinib (Zhang et al., 2023a).

###### 3.1.3.3.2 Regulation mechanisms

Hypoxia increases CCL22 expression in TAMs, which significantly boosts the migration and lung metastasis of TNBC cells via the CCL22/CCR4 axis (Zhao et al., 2022). CCL22 expression by macrophages is modulated through the IL-4/STAT6 signaling pathway, which is pivotal for T cell differentiation into Th2 cells and crucial for CCL22 and VEGF-C expression in tongue squamous cell carcinoma, contributing to lymph node metastasis (Kimura et al., 2021). In osteosarcoma, M2 macrophages promote cell migration and EMT, with CCL22 expression being notably high in LINC00662 exosome-treated M2 macrophages. The neutralization of CCL22 using a specific antibody has been shown to reverse these pro-tumorigenic effects, suggesting a therapeutic approach to mitigating cancer progression by targeting CCL22 (Zhang Y. et al., 2023). Additionally, TAM-secreted TGF-β induces CCL22 expression via c-Fos, facilitating the recruitment of regulatory T cells and establishing a feedback loop with IL-8 and TGF-β that enhances CCL22 secretion in TAMs (Wang et al., 2019).

###### 3.1.3.3.3 Therapeutic potential

CCL22 also plays a crucial role in CRC chemoresistance by activating the PI3K/Akt pathway, thereby reducing the effectiveness of 5-fluorouracil in curbing tumor growth (Wei et al., 2019b).

### 3.2 Interaction with CAFs

CAFs, a distinct group within the tumor stroma, play a crucial role in facilitating EMT and the metastatic spread of cancer (Yang et al., 2022). TAMs interact with CAFs in numerous ways, significantly influencing tumor behavior and treatment response (Gunaydin, 2021b). A noteworthy interaction involves TAMs secreting CCL18, which engages CAFs expressing CD10 and GPR77. This interaction is associated with the promotion of CSC characteristics and the development of chemotherapy resistance in BC (Zeng et al., 2022).

#### 3.2.1 OSM-OSMR signaling

OSM-OSMR signaling pathway is crucial in the reprogramming of CAFs, thereby significantly impacting tumor metastasis, particularly in PDAC. OSM, produced by macrophages, induces CAFs to secrete IL-6 and CCL2. These cytokines then activate key signaling pathways, specifically the PI3K/Akt and Jak/STAT pathways, which play a critical role in promoting EMT (Lee et al., 2021). Furthermore, CAFs mitigate the pro-inflammatory effects of the chemotherapy regimen FOLFIRINOX on CD200R+CD209+ macrophages, limiting cell death and reinforcing the M2 phenotype of TAMs (Hussain et al., 2024).

#### 3.2.2 Reciprocal interactions and recruitment

The interplay between TAMs and CAFs is reciprocal, with the influence of CAFs on TAMs becoming increasingly apparent. CAFs actively recruit macrophages to the TME by secreting a variety of chemokines, leading to a significant co-infiltration of CAFs and TAMs, which is often correlated with tumor progression. In OSCC, for instance, CAFs have been observed to recruit monocytes via the CXCL12/CXCR4 axis (Li et al., 2019). Additionally, CAFs can induce an M2-like polarization in TAMs, with both tumor-conditioned and CAF-conditioned mediums synergistically enhancing the expression of CD206 and IL-10 in TAMs. This synergistic effect is largely attributed to factors secreted by CAFs, notably GM-CSF and IL-6 (Cho et al., 2018). Moreover, CAFs also have the capability to elevate ROS levels in TAMs, further promoting their polarization toward an M2 phenotype (Pakravan et al., 2022). Furthermore, TAMs establish crucial interactions with CAFs through the CD74/macrophage migration inhibitory factor (MIF) pathway. This interaction is pivotal for the recruitment and polarization of TAMs within the TME. In addition, TAMs plays a significant role in attracting regulatory B cells (Bregs) that express PD-L1, utilizing the CXCL12/CXCR4 axis (Lian et al., 2024).

#### 3.2.3 Transformation of noncancerous fibroblasts

Mixed-polarized macrophages, expressing both M1 and M2 markers, activate noncancerous fibroblasts (NFs) to transform into cancer-associated fibroblast-like (CAF-like) cells, which in turn promote the malignant phenotype of diffuse-type gastric cancer (DGC) cells. IL-1β, released by these mixed-polarized macrophages, triggers NF-kB signaling in NFs, catalyzing their activation and subsequent transdifferentiation into CAF-like cells. This process is evidenced by the nuclear translocation of p65 and a surge in the expression of fibroblast activation protein alpha (FAP) (Zhang et al., 2023c).

#### 3.2.4 TAM-endothelial cell (EC) interaction

**FIGURE 3:**
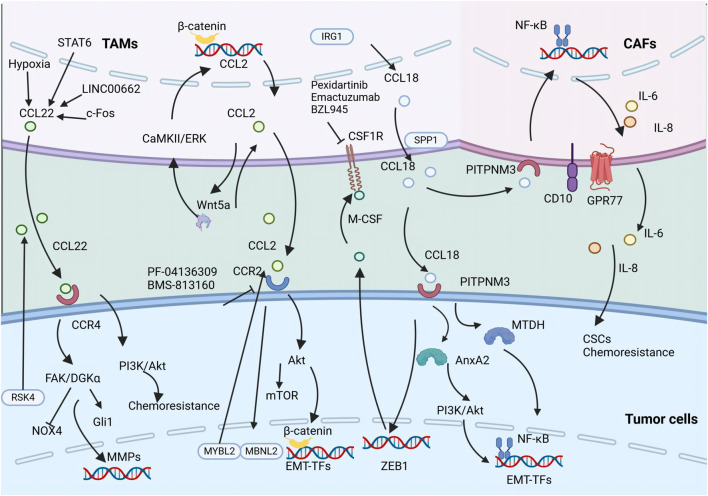
The secretion of various chemokines including CCL18, CCL2, and CCL22 by TAMs promotes tumor metastasis and CSC properties. The illustration depicts the signaling cascade of TAM-secreted CCL18, CCL2, and CCL22 that promotes EMT and augments the stemness of neoplastic cells.

Moreover, the reciprocal interaction between TAMs and ECs is critical within the TME, especially in facilitating intratumoral angiogenesis. Recent advancements in biomimetic 3D models have shown that TAM-EC communication relies on direct contact, with M2-like TAMs increasing the secretion of anti-inflammatory cytokines, thereby fostering invasion in glioblastoma (Figure 3) (Cui et al., 2018).

## 4 TAM-induced EMT: Shaping cancer diversity

The intersection of TAMs with the EMT process underscores a fundamental aspect of tumor biology, revealing a vast heterogeneity across various cancers (Feng et al., 2022). In this analysis, we aim to dissect the complex relationships between TAMs and EMT within four specific cancer contexts, each chosen for its unique clinical challenges and the significant role TAMs play in their progression.

1. TNBC is characterized by its aggressive nature and a conspicuous lack of targeted therapies. This section will elucidate the contribution of TAMs to TNBC progression, with a particular focus on their involvement in EMT mechanisms.
2. The resistance to EGFR-TKI in lung cancer represents a significant clinical hurdle. Our discussion will center on how TAMs influence this resistance, potentially through EMT modulation.
3. The fibrotic microenvironment, a hallmark of pancreatic cancer, is significantly shaped by pro-fibrotic cytokines such as TGF-β and OSM, which are instrumental in the pathology of this malignancy. We aim to explore the impact of TAMs within this context, specifically their influence on EMT processes.
4. Androgen deprivation therapy (ADT) is a fundamental component of prostate cancer treatment, yet its long-term efficacy remains limited. This investigation will focus on how TAMs interact with the prostate cancer microenvironment in the context of ADT, particularly regarding their influence on EMT and disease progression.

### 4.1 Breast cancer——Entry point for TNBC

TNBC is a particularly aggressive subtype of BC, characterized by the absence of estrogen receptors, progesterone receptors, and the human epidermal growth factor receptor 2 (HER2). This lack of receptors renders TNBC without specific targets for established therapies, significantly complicating treatment efforts. Consequently, there has been a heightened focus on exploring the interactions between TAMs and TNBC cells to identify new therapeutic avenues. Given the critical role TAMs play in TNBC progression, macrophage-targeting immunotherapy emerges as a promising strategy (Fernandez et al., 2023).

#### 4.1.1 Epigenetic reprogramming of TAMs

In TNBC, TAMs compared to BMDMs and MGMs showed distinct DNA methylation patterns. These methylation patterns retain imprints from their monocytic origins but undergo cancer-specific epigenetic changes. Key transcription factors such as FOSL2, STAT1, and RUNX3 are involved in this epigenetic reprogramming, which correlates with more severe tumor grades and poorer prognoses in breast cancer patients (Hey et al., 2023). Additionally, a significant negative correlation exists between the TAM marker CD163 and E-cadherin expression in TNBC, indicating that higher TAM presence is associated with worse patient outcomes (Zhang et al., 2018).

#### 4.1.2 Chemokine-mediated EMT

TAM-secreted chemokines, particularly within the CXCL8/CXCR2 axis, play a crucial role in facilitating EMT processes in TNBC. The antagonism of CXCR2, for example, with Danirixin, presents a potential therapeutic strategy to counteract this mechanism (Nie et al., 2021). Additionally, Additionally, CCL2 released by M2 macrophages activates β-catenin, enhancing cancer stemness and promoting the EMT process in TNBC (Chen X. et al., 2022). This CCL2/β-catenin signaling loop highlights the complex interplay between TAM signaling and TNBC progression (Zhang F. et al., 2021). Furthermore, the endogenous protein visfatin, produced by TNBC cells, recruits and polarizes macrophages. These TAMs subsequently augment tumor cell mobility and self-renewal capacity through the secretion of CXCL1 (Wang et al., 2020).

#### 4.1.3 VEGFA and cancer stemness

TAMs contribute significantly to the complexity of the TME in TNBC by secreting VEGFA. This secretion interacts with TNBC cells through the neuropilin-1 (NRP-1) receptor, activating the GAPVD1/Wnt/β-catenin signaling pathway, thereby enhancing the cancer stemness of TNBC cells. This process fuels tumor progression and metastasis while also promoting the further polarization of macrophages towards the M2 pro-tumorigenic phenotype. The release of VEGFA by M2-like TAMs and its subsequent actions establish a feedback loop that exacerbates the metastatic capabilities of TNBC (Wang L. et al., 2024).

#### 4.1.4 Hippo-YAP pathway

The Hippo signaling pathway, particularly the YAP, is aberrantly expressed in both TAMs and TNBC cells, emerging as a pivotal player (Xu et al., 2022; Yang et al., 2020). Research reveals that TNBC cells can upregulate YAP expression in TAMs through otu deubiquitinase 5 (OTUD5)-dependent deubiquitination and stabilization of YAP. This overexpression leads to M2 macrophage polarization and enhances metastatic capabilities via the CCL2/CCR2 pathway in TNBC (Zhang et al., 2021c). Furthermore, the activation of the Hippo-YAP pathway in TNBC cells, driven by RAD18, stimulates cancer cell proliferation and invasion. This pathway activation also correlates with increased expression of the M2 macrophage marker CD163 in TAMs, further underlining the contribution of the Hippo-YAP axis to TNBC progression (Yang et al., 2020). In a reciprocal manner, M2-polarized TAMs secrete TGF-β to activate RAD18, thus promoting cancer stemness and establishing a feedback loop. Disrupting this loop can suppress cancer stemness, highlighting the critical interplay between RAD18, YAP, and TGF-β in TNBC CSC characteristic (Yan et al., 2022).

#### 4.1.5 GLUT3 and glycolytic activity

Recent studies have underscored the significant role of the GLUT3 in TNBC metastasis. The endogenous expression of GLUT3 directly promotes the EMT process. When GLUT3 expression is upregulated, it enhances the glycolytic activity of TNBC cells, leading to increased lactate production. This lactate stimulates TNBC cells to secrete CXCL8, inducing a phenotype in macrophages that resembles M1-like pro-inflammatory macrophages. Once activated, these M1-like macrophages contribute to further upregulation of GLUT3 expression, creating a feedback loop that sustains high glycolytic activity and EMT in TNBC cells (Tsai et al., 2021).

#### 4.1.6 Targeted therapy innovations

The mannose receptor serves as a crucial target for directing therapies towards TAMs in TNBC. Innovative theranostic nanoformulations leverage the interactions between mannose and its receptor to specifically target TAMs in the acidic environment characteristic of TNBC tumors. These nanoformulations are engineered with an acid-sensitive polyethylene glycol (PEG) layer that sheds to reveal mannose ligands upon exposure to the acidic TME. One such application involves DOX (doxorubicin)-loaded nanovehicles, which have shown significant promise in targeting TAMs and improving therapeutic outcomes (Scialla et al., 2023). Additionally, cutting-edge nanocomplexes designed to react to the acidic and oxidative tumor environment can facilitate the release of miR-155. This release encourages a shift towards an M1-like phenotype in TAMs and fosters the maturation of tumor-infiltrating dendritic cells (TIDCs), thereby amplifying the anti-tumor immune responses in TNBC (Jing et al., 2023).

#### 4.1.7 Role of MGRTMs in early TNBC

Notably, research has identified that in the early stages of TNBC, the pro-tumoral macrophages are predominantly mammary gland tissue-resident macrophages (MGRTMs), rather than monocyte-derived TAMs. MGRTMs, characterized as F4/80+FOLR2+CD206+CADM1-, stimulate *in vitro* proliferation and enhance *in vivo* angiogenesis. Local depletion of MGRTMs has been shown to reduce metastatic potential (Hirano et al., 2023b).

#### 4.1.8 Inflammatory breast cancer (IBC)

IBC, an exceptionally malignant form of BC, is characterized by its aggressive nature, stem cell-like traits, and high propensity for metastasis, distinguishing it from other subtypes (Lim et al., 2018). Co-cultivation of IBC cells with monocytes significantly enhances CSC characteristics. IBC cells attract and differentiate monocytes into M2 TAMs, which release large amounts of IL-8 and growth-related oncogene (GRO). This release activates the STAT cascade in IBC cells, further exacerbating the malignant phenotype (Valeta-Magara et al., 2019).

### 4.2 Lung cancer——EGFR-TKIs and oncometabolites

#### 4.2.1 Role of TAMs in EGFR-TKI resistance

In NSCLC, particularly in adenocarcinoma subtypes common among non-smokers or light smokers, mutations in the epidermal growth factor receptor (EGFR) are a primary oncogenic driver (Vyse and Huang, 2019). Emerging evidence implicates TAMs in facilitating the EMT process in NSCLC, thereby contributing to resistance against EGFR-TKIs. Specifically, in the context of EGFR-TKI resistance, TAMs exhibit M2-like reprogramming and reduced phagocytosis. This reprogramming correlates with the resistance observed in gefitinib-resistant lung cancer cells and tumor xenografts (Lu et al., 2023).

#### 4.2.2 Activation and recruitment of TAMs

The activation of EGFR signaling in tumor cells initiates a cascade that leads to the recruitment of TAMs, which subsequently promote the upregulation of EMT-TFs in NSCLC (Ravi et al., 2016; Zhu et al., 2019). An intriguing finding relates to the dual effect produced by the activation of cannabinoid receptor 2 (CB2) through the compound JWH-015. This activation appears to counteract the pro-tumorigenic signaling mediated by M2-like TAMs, specifically inhibiting EGFR/ERK/STAT3 signaling and N-cadherin expression, both promoted by M2-like TAMs. Moreover, JWH-015 reduces ARG1 expression in TAMs (Ravi et al., 2016).

A recent study has highlighted the role of TAM-derived IL-6, particularly in EGFR mutant NSCLC, revealing that IL-6 can augment the immunotherapy resistance of tumor cells with EMT-associated resistance to EGFR-TKI (Wang et al., 2023). Significantly, TAMs have been identified as the primary source of IL-6, underscoring their crucial role in the resistance mechanism and highlighting potential therapeutic targets to overcome resistance in EGFR-TKI therapy.

#### 4.2.3 Phagocytic checkpoints

The Mer receptor tyrosine kinase (MERTK) present on macrophages acts as a phagocytic checkpoint and is associated with EGFR-TKI resistance in NSCLC. Elevated MERTK expression in both tumor cells and macrophages contributes to TKI resistance by promoting cell polarity and stemness in tumor cells while sending 'do not eat me’ signals that help maintain an immunosuppressive TME (Chen and Liu, 2021). Another critical phagocytic checkpoint is SIRPα. TAMs express high levels of SIRPα, which is positively correlated with IL-6 expression. The SIRPα-IL-6 axis forms a self-reinforcing feedback loop, where each component upregulates the other through STAT3 signaling in macrophages (Wang et al., 2023).

#### 4.2.4 Resistance to osimertinib

Resistance to the third-generation EGFR-TKI, osimertinib, presents a formidable challenge in treating certain cancers. Research has highlighted the roles of IL-6, the EMT process, and TAM activity in contributing to this resistance. Studies utilizing RNA sequencing and immune infiltration analysis have revealed an association between increased macrophage infiltration, particularly M0 and M2 macrophages, and resistance to osimertinib in patients (Han et al., 2023). The EMT process in tumor cells has been identified as a key mechanism behind osimertinib resistance. The involvement of TAMs in inducing EMT underscores a potential therapeutic target; by mitigating TAM-induced EMT, it may be possible to overcome resistance to osimertinib and potentially other TKIs (Jiang et al., 2022; Yan et al., 2023).

#### 4.2.5 Innovative approaches

One mechanism by which tumor cells develop TKI resistance involves the upregulation of CD47, helping them evade macrophage-mediated phagocytosis—a key way macrophages can contribute to overcoming drug resistance (Lu et al., 2023). Innovative approaches, including the use of STING agonists and anti-CD47 monoclonal antibodies, are being explored to reprogram TAMs to combat this resistance effectively (Lu et al., 2023). TAMs can suppress IFNγ and Granzyme B production in CD8 T cells. MSA-2, a STING agonist, triggers IFNβ production in TAMs, further emphasizing the importance of macrophages in the STING-mediated antitumor response (Lin Z. et al., 2023).

#### 4.2.6 Metabolic interplay and resistance

The metabolic interplay between TAMs and tumor cells significantly influences osimertinib resistance. Succinate, an intermediate in the TCA cycle, has been highlighted for its role in TAM polarization and EMT induction in lung cancer through activation of the succinate receptor 1 (SUCNR1) and subsequent triggering of the PI3K/HIF-1α pathway. This promotes M2 polarization of TAMs and EMT in tumor cells (Wu J.-Y. et al., 2020). Additionally, targeting lipid metabolism with simvastatin has emerged as a potential therapeutic approach. By disrupting critical pathways involved in TAM polarization and EMT, simvastatin can restore sensitivity to chemotherapy and offer a new strategy to combat resistance in EGFR-TKI-treated NSCLC (Jin et al., 2019).

### 4.3 Pancreatic cancer——Profibrotic cytokines

Pancreatic cancer, known for its aggressive behavior and dire prognosis, is characterized by a distinct microenvironment heavily marked by fibrosis (Pratt et al., 2021). This fibrotic milieu is largely governed by key profibrotic cytokines such as TGF-β and OSM, which are central to the disease’s pathology. Within this fibrotic landscape, TAMs stand out as a predominant cell type, exerting a significant influence on cancer progression through their interaction with these cytokines and their downstream signaling pathways (Stawski and Trojanowska, 2019; Maria et al., 2022).

#### 4.3.1 TAM modulation of TGF-β signaling

TAMs are recognized for their role in modulating the TGF-β signaling pathway, particularly through the production of miR-501-3p. This microRNA regulates TGF-β signaling by inhibiting TGFBR3 and upregulating TGFBR1, TGFBR2, and phosphorylated SMAD3, thereby amplifying TGF-β signaling and promoting EMT (Yin et al., 2019). Research indicated that treatment with TAM-CM can escalate the expression of EMT markers such as Snail, Vimentin, and N-cadherin in PDAC cells, while reducing E-cadherin expression and augmenting SMAD3 phosphorylation. The effectiveness of TGF-β neutralizing antibodies in inhibiting TAM-induced EMT further underscores the pivotal role of TAM-derived TGF-β in PDAC progression (Xiong et al., 2021).

The TGFβ vaccine has demonstrated efficacy in PDAC by decreasing the proportion of M2-like TAMs and influencing the polarization of CAFs away from the myofibroblast-like phenotype. This phenotype is associated with the development of a rigid extracellular matrix that impedes T cell infiltration into tumors. T cells specific to TGF-β, stimulated by the vaccine, can alter fibroblast phenotypes, reducing the expression of myofibroblast-associated markers such as alpha-smooth muscle actin (αSMA) and transgelin (TAGLN). By targeting and modifying TAMs and CAFs, the TGF-β vaccine aims to alleviate immunosuppression and circumvent immune exclusion within pancreatic tumors. This strategic modulation of the TME seeks to make PDAC more responsive to immune-based therapies, potentially enhancing the effectiveness of such treatments (Perez-Penco et al., 2024).

Additionally, TAMs characterized by CD51 expression, indicative of M2 polarization, have been found to promote CSC characteristics in PDAC by releasing TGF-β1. This cytokine activates SMAD2/3 signaling in cancer cells, leading to an upregulation of stemness markers like Nanog, Sox2, and Oct3/4, thereby contributing to the complexity of PDAC progression (Zhang et al., 2019).

#### 4.3.2 OSM-OSMR signaling in PDAC

OSM plays a crucial role in TAM-tumor interactions by binding to its receptor, OSMR, on tumor and stromal cells, activating various signaling pathways, including Jak-STAT (West, 2019). Elevated OSMR expression in PDAC cells is associated with poor patient outcomes (Smigiel et al., 2017).

TAMs are capable of secreting OSM, which can activate the OSM-OSMR signaling pathway in neighboring mesenchymal cells, specifically CAFs. This cascade promotes the expression of proinflammatory cytokines in CAFs, establishing a feedback loop with TAMs and tumor cells that facilitates tumor metastasis (Lee et al., 2021).

Recent studies using patient-derived xenograft models of PDAC have shown that TAM-secreted OSM can enhance EMT. This is evidenced by increased expression of LOXL2 and Snail, with LOXL2 reduction linked to decreased metastasis and CSC traits in PDAC (Alonso-Nocelo et al., 2022). Targeting the OSM-LOXL2 signaling pathway with LOXL2 inhibitors, possibly in combination with TAM-targeting therapies, may offer a new strategy to curb PDAC metastasis (Alonso-Nocelo et al., 2022).

#### 4.3.3 IL-1β+ TAMs and inflammatory loop

IL-1β+ TAMs play a pivotal role in the progression of PDAC by participating in a reciprocal inflammatory loop with PDAC cells. Exposure to pro-inflammatory stimuli like PGE2 and TNF induces these TAMs to polarization, which not only reprograms adjacent PDAC cells but also boosts the production of PGE2, TNF, and other factors, sustaining the IL-1β TAM phenotype. IL-1β+ TAMs are crucial in PDAC progression, forming a reciprocal inflammatory loop with PDAC cells (Caronni et al., 2023).

#### 4.3.4 Gemcitabine resistance

Besides, TAMs are involved in gemcitabine resistance. Tissue-resident macrophages within PDAC endure chemotherapy by increasing deoxycytidine (dC) production while reducing deoxycytidine kinases (dCKs) levels, thereby decreasing gemcitabine uptake (Zhang et al., 2023d). Nonetheless, targeting the myeloid spleen tyrosine kinase (Syk) in macrophages can induce a shift towards an immunostimulatory phenotype. The use of the FDA-approved Syk inhibitor R788 has been shown to transform the tumor immune microenvironment, reprogramming pro-tumorigenic macrophages into an immunostimulatory state. This reprogramming significantly bolsters CD8^+^ T-cell activity, thereby enhancing the therapeutic efficacy of gemcitabine (Rohila et al., 2023).

### 4.4 Prostate cancer——Androgen deprivation therapy

Prostate cancer, a prevalent malignancy among men, is closely associated with the inflammatory TME in terms of its development and progression. A notable aspect of prostate cancer treatment involves ADT, which, despite its initial effectiveness, often leads to resistance and disease progression. This section explores the role of TAMs in this context, particularly their influence on the EMT process and therapeutic resistance.

#### 4.4.1 TAM-induced chemokines secretion

In 2013, a key discovery revealed that TAMs can stimulate the secretion of CCL4 through the activation of the macrophage androgen receptor (AR). This activation triggers STAT3 and initiates EMT in prostate tumor cells, downregulating tumor suppressors like P53/PTEN and raising levels of EMT markers such as Snail and MMP9. The utilization of anti-CCL4 neutralizing antibodies presents a potential strategy to counteract TAM-mediated EMT and metastasis (Fang et al., 2013).

Further, TAM-derived CCL5 contributes significantly to metastasis and chemotherapy resistance. Elevated CCL5 expression in prostate cancer enhances tumor cell migration and invasion, upregulating N-cadherin, MMP2, MMP9, and the CD44^+^CD133+ cell subpopulation. Neutralizing CCL5 can inhibit these effects, suggesting a potential intervention strategy (Huang et al., 2020). Furthermore, CCL5 derived from TAMs can induce STAT3 activation, facilitating the EMT process and CSC phenotype. Inhibiting STAT3 signaling can attenuate chemoresistance and inhibit lung metastasis in prostate cancer (Ma et al., 2021).

#### 4.4.2 ADT resistance

ADT is an established first-line therapy for managing advanced prostate cancer (Cooperberg et al., 2003). However, suppression of AR function has been linked to increased TAM infiltration, fostering EMT and metastasis (Cooperberg et al., 2003). Recent studies highlight the critical role of macrophages in the bone-metastatic prostate cancer microenvironment. Macrophages promote resistance to ADT through the cytokine activin A, which triggers the fibronectin (FN1)-integrin alpha 5 (ITGA5)-tyrosine kinase Src (SRC) cascade. Genetic models show that both bone-resident and monocyte-derived macrophages are essential for enzalutamide resistance, a common anti-androgen therapy (Li X. F. et al., 2023).

Moreover, cathepsin K (CTSK) expression, linked to M2 TAM infiltration, is upregulated in castration-resistant prostate cancer (CRPC) and may activate IL-17 signaling, promoting metastasis (Wu N. et al., 2022). ADT treatment also inhibits the SAM-pointed domain-containing ETS transcription factor (SPDEF), increasing CCL2 expression and leading to EMT and ADT resistance (Tsai et al., 2018). Inhibition of AR function significantly promotes CCL2 expression and secretion in TAMs, inducing EMT. Neutralizing CCL2 attenuates STAT3 cascades and inhibits the EMT process. Moreover, inhibiting both AR and CCL2/CCR2 signaling could reduce tumor progression *in vivo* (Izumi et al., 2013). These findings highlight the significant role of CCL2-mediated TAM-tumor interactions in promoting EMT and resistance to therapeutic interventions in the context of ADT for prostate cancer.

#### 4.4.3 NOTCH signaling

Direct contact between macrophages and tumor cells in prostate cancer promotes the EMT process. The NOTCH signaling pathway, which relies on the physical interaction between a receptor and its ligand, is pivotal in this context (Siebel and Lendahl, 2017). When TAMs interact directly with prostate cancer cells, downstream factors associated with NOTCH1 signaling are significantly upregulated. This contact enhances the expression of genes related to EMT. Inhibiting TAMs phenotype or NOTCH1 signaling could be a viable strategy to impede prostate cancer progression (Shi F. et al., 2022).

## 5 Clinical implications

### 5.1 Targeting TAM infiltration

Therapeutic strategies aimed at curbing TAM infiltration have garnered significant interest as a potential avenue for cancer treatment. The focus on pharmacological agents that can selectively target TAMs has led to the development and clinical evaluation of various compounds, with some showing promising results in reducing TAM-mediated pro-tumorigenic functions.

#### 5.1.1 M-CSF/CSF-1R

M-CSF, a crucial cytokine in macrophage regulation, plays a vital role in the differentiation, survival, and functional activity of macrophages. Its signaling pathway is implicated in TAM recruitment and the promotion of their pro-tumorigenic activities. Targeting M-CSF or its receptor CSF-1R has emerged as a promising strategy (Mun et al., 2020).

Pexidartinib (PLX3397), a small molecule inhibitor of CSF-1R, has shown efficacy in both preclinical studies and clinical trials. PLX3397 induces cell death in tenosynovial giant cell tumors (TGCT), which are highly infiltrated by M-CSF-dependent macrophages (Thongchot et al., 2023). Additionally, PLX3397 treatment reduces M2 polarization and TAM proliferation in sarcomas, blocks tumor metastasis, and improves lymphocyte infiltration in an orthotopic osteosarcoma mouse model (Fujiwara et al., 2021). Mechanistically, PLX3397 disrupts TAM recruitment and inhibits CCL22 release, thereby impairing TAM-mediated immune suppression and metastasis promotion (Zhang et al., 2023a). Notably, this drug has also been shown to inhibit the infiltration of microglia in the brain while sparing peripheral macrophages, highlighting its potential for targeting central nervous system (CNS) tumors without significantly impacting peripheral immune functions (Okojie et al., 2023).

A phase 3 clinical trial evaluating the efficacy of PLX3397 in the treatment of TGCT demonstrated promising results in terms of ameliorating patient symptoms and improving overall outcomes in adults with TGCT (NCT02371369). Nonetheless, careful attention must be paid to the potential hepatotoxicity associated with this therapeutic agent (Tap et al., 2019). Currently, the further safety and efficacy assessment of PLX3397 remains ongoing (NCT04635111, NCT04488822, NCT04635111). Recently, there has also been attention focused on the targeted delivery and synergistic therapy of PLX3397 (Liang et al., 2023; Zhang et al., 2023e).

Emactuzumab (RG7155), an anti-CSF1R monoclonal antibody, reduces the quantity and functional activity of TAMs in various cancer types and promotes CD8^+^ T cell infiltration. Additionally, RG7155 significantly decreases the population of CSF-1R+CD163+ TAMs in patients with diffuse-type giant cell tumors (Ries et al., 2014). However, under certain circumstances, TAMs may evade RG7155 therapy by relying on alternative factors, such as IL-4 (Pradel et al., 2016a). Therefore, combining a CSF-1R inhibitor with IL-4 signaling blockade could be a promising therapeutic approach. RG7155 has undergone phase 1 clinical evaluation in advanced solid tumor patients. The trial, involving 217 patients, assessed the safety, tolerability, pharmacokinetics, pharmacodynamics, and preliminary efficacy of RG7155. Results indicated that RG7155 has a favorable tolerability profile and clinical efficacy in inhibiting M2 TAMs (NCT01494688) (Gomez-Roca et al., 2019). Additionally, combining RG7155 with the CD40 agonist selicrelumab significantly improves the immune microenvironment in solid tumors, notably increasing CD8^+^ T cell infiltration and showing effectiveness when used in combination with atezolizumab (Machiels et al., 2020; Gomez-Roca et al., 2022).

BLZ945, a small inhibitor of CSF-1R, has shown promise in research studies. It can inhibit tumor progression by reducing TAM and MDSC infiltration in HCC (He et al., 2021). Additionally, BLZ945 impedes the M2 polarization of TAMs and reduces the tumor burden in OSCC (Guo et al., 2020). A phase I clinical trial evaluating BLZ945 alone or with PDR001 for advanced solid tumors demonstrated favorable safety and tolerability profiles (NCT02829723). Recent study has shown that BLZ945 exerts immunomodulatory effects both in the periphery and within the TME of solid tumors, including inhibiting monocyte infiltration and promoting T cell infiltration (Alcazar et al., 2024).

CSF-1R inhibitors like PLX3397 and RG7155 offer several advantages and disadvantages in cancer treatment. These inhibitors are known for their target specificity, allowing them to specifically target macrophages while reducing off-target effects and sparing other immune cells, thereby minimizing unintended side effects. Additionally, they have significant combination potential, as they can be used with other therapies, such as chemotherapy and checkpoint inhibitors, to enhance overall treatment efficacy (Gomez-Roca et al., 2019). By reducing TAM populations, these inhibitors can modulate the immune system, potentially reversing immunosuppression within the tumor microenvironment and enhancing anti-tumor immune responses.

However, there are notable disadvantages associated with CSF-1R inhibitors. Toxicity, particularly hepatotoxicity, is a concern, necessitating careful monitoring and dose adjustments during treatment (Lewis et al., 2021; Viganò et al., 2023). Tumors may also develop resistance mechanisms by upregulating alternative pathways, such as IL-4 (Pradel et al., 2016a), to maintain TAM populations, which can limit the long-term efficacy of these treatments. Furthermore, while preclinical data is promising, more extensive clinical trials are required to fully establish the safety and efficacy profiles of CSF-1R inhibitors.

#### 5.1.2 CCL2/CCR2

The CCL2/CCR2 signaling pathway represents a pivotal mechanism that governs the recruitment and activation of TAMs within the TME. Interventions that selectively target this pathway have demonstrated considerable promise in impeding the process of EMT mediated by TAMs.

PF-04136309, a specific CCR2 inhibitor, significantly reduces inflammatory TAM infiltration and inhibits tumor metastasis in preclinical PDAC models. It effectively suppresses macrophage infiltration in both primary tumors and liver metastases (Yang and Zhang, 2017). Combining PF-04136309 with a CXCR inhibitor suppresses TAM and TAN infiltration in PDAC, enhancing T cell-mediated anti-tumor responses. (Nywening et al., 2018). Additionally, PF-04136309 inhibits GOLM1-mediated MDSC infiltration, reducing CRC metastasis (Dang et al., 2021).

A phase 1b study evaluated PF-04136309 combined with FOLFIRINOX chemotherapy, it shows a higher rate of tumor response and local tumor control compared to FOLFIRINOX alone, although it is associated with significant adverse events, particularly neutropenia and hypokalemia (Wang-Gillam et al., 2015; Nywening T. et al., 2016). Another phase 1b clinical study established the safety and feasibility of combining PF-04136309 with nab-paclitaxel and gemcitabine for treating metastatic PDAC. Importantly, the treatment led to a decrease in CD14^+^ CCR2+ inflammatory monocytes in the peripheral blood, although these cells did not accumulate in the bone marrow (Noel et al., 2020a).

BMS-813160, a recently developed small molecule, impedes the binding of chemokines CCL2 and CCL5 to their receptors, CCR2 and CCR5. It is highly potent and selective, with IC50 values of 6.2 and 3.6 nM, respectively. BMS-813160 also has favorable pharmacokinetic properties, including good permeability, stability, oral bioavailability, and low clearance in animal models (Tu et al., 2020). An ongoing phase 1b/2 clinical trial is investigating BMS-813160 combined with chemotherapy or nivolumab for treating metastatic colorectal or pancreatic cancers (NCT03184870). And BMS-813160 reduces the recruitment and activity of MDSCs and regulatory T cells, thereby diminishing the immunosuppressive milieu and potentially enhancing antitumor immune responses (Le et al., 2018). Besides, a phase IIa trial indicates that while BMS-813160 and BMS-986253 (anti-IL8) show biological activity by affecting chemokine levels in NSCLC and HCC, reducing the recruitment of circulating monocytes to the TME (Venturini et al., 2023).

Despite the promising results from preclinical and early-phase clinical studies, there are still limitations. More extensive trials are needed to fully establish the long-term safety and efficacy of these therapies. Regarding the adverse events associated with PF-04136309, a significant number of patients experienced grade 3 or higher neutropenia when combined with FOLFIRINOX. This condition involves a low count of neutrophils, which are essential for fighting infections (Nywening T. M. et al., 2016). Additionally, when PF-04136309 was used in combination with nab-paclitaxel and gemcitabine, there was a high incidence (24%) of pulmonary toxicity. This raises concerns about the safety profile of PF-04136309 in such combinations (Noel et al., 2020a).

In addition to BMS-813160 and PF-04136309, several other CCR2 inhibitors have shown promising potential in cancer therapy. CCX872-B has demonstrated efficacy in combination with chemotherapy for PDAC by reducing immunosuppressive myeloid cell recruitment (Noel et al., 2017). MLN1202, a monoclonal antibody targeting CCR2, has been explored in bone metastasis of cancer and inflammatory conditions, effectively blocking the migration of CCR2+ cells into the TME (Yumimoto et al., 2019). RS504393, a non-peptide CCR2 antagonist, has shown preclinical success in inhibiting myeloid cell recruitment (Mu et al., 2019). These compounds highlight the ongoing advancements in targeting CCR2 to enhance cancer treatment outcomes.

### 5.2 Targeting cytokine and growth factor signaling

TAMs secrete a diverse array of cytokines and growth factors that facilitate the progression of tumor growth, invasion, and metastasis. Interfering with these signaling molecules has demonstrated considerable potential in restraining TAM-mediated EMT, thereby serving as a propitious therapeutic approach.

#### 5.2.1 TGF-β

TGF-β promotes tumor growth and metastasis by inducing EMT. Several drugs have been developed to inhibit TGF-β signaling and suppress TAM-induced EMT.

Galunisertib, a selective TGFBR1 inhibitor, has shown promising preclinical results, combining galunisertib with PD-L1 blockade enhanced tumor growth inhibition (Holmgaard R. et al., 2018). It inhibits macrophage infiltration and M2 polarization *in vivo* in prostate cancer models and attenuates TAMs-derived CXCL5 mediated EMT process (Wu et al., 2022b). Galunisertib is currently under evaluation in clinical trials as a monotherapy or in combination with chemotherapy or immunotherapy for treating advanced solid tumors, including HCC (Kelley et al., 2019; Faivre et al., 2019), metastatic PDAC (Melisi et al., 2021a) and locally advanced rectal cancer (Yamazaki et al., 2022). However, in a trial involving 32 patients with metastatic pancreatic cancer, only one patient experienced a partial response, and the disease control rate was 25.0%, highlighting limited clinical activity (NCT02734160) (Melisi et al., 2021b).

Fresolimumab, a human monoclonal antibody that neutralizes all three isoforms of TGF-β, has yet to undergo comprehensive preclinical investigation regarding its association with TAMs (Peng et al., 2022). Higher dose group exhibited a stronger systemic immune response, evidenced by increased peripheral blood mononuclear cells and an expansion of the CD8^+^ T cell central memory pool (Formenti et al., 2018). It has been examined in both phase 1 and 2 clinical trials. A phase 1 trial in patients with malignant melanoma and renal cell carcinoma showed that fresolimumab had acceptable safety profiles and demonstrated antitumor efficacy, though some adverse events were reported (Morris et al., 2014). A phase 2 trial in patients with NSCLC is currently ongoing (NCT02581787). Additionally, there have been instances where fresolimumab treatment led to the development of skin lesions resembling benign keratoacanthomas, and in one patient with a history of skin cancer, there was a potential link to the development of squamous cell carcinoma (Arjaans et al., 2012).

#### 5.2.2 IL-6

IL-6 plays a vital role in orchestrating EMT, facilitating neoplastic progression and promoting immune-related adverse events (irAEs). Multiple pharmacological agents have been evaluated for their efficacy in blocking IL-6 or its downstream signaling cascades to impede TAM-induced EMT and suppress malignant growth.

Tocilizumab, a monoclonal antibody targeting the IL-6 receptor, has been extensively investigated. Many preclinical studies show its potential in inhibiting TAM-induced EMT and mitigating cancer progression. In 2014, it was found that tocilizumab inhibits CSC characteristics induced by TAMs by blocking IL-6/STAT3 signaling in HCC. *In vitro* and *in vivo* studies showed that tocilizumab significantly reduced the CD44^+^ subset (Wan et al., 2014a). In BC, tocilizumab attenuated the MCT-1/IL-6/IL-6R signaling between tumor cells and TAMs, inhibiting stemness (Weng Y. et al., 2019).

IL-6, a pro-inflammatory cytokine, has recently gained attention for its role in reducing immunotherapy toxicity when combined with immune checkpoint blockade (ICB) therapy. In patients and mice receiving ICB therapy, IL-6 expression, neutrophil infiltration, and chemotactic markers increased in inflamed tissues (Holmstroem et al., 2022). An open-label study showed that tocilizumab effectively treated ICB-induced colitis and arthritis with manageable safety profiles (Holmstroem et al., 2022). Tocilizumab treatment reduced the severity and frequency of irAEs in patients and mice receiving ICB therapy without compromising the anti-tumor immune response. This shift was due to blocking IL-6, changing the immune balance in the TME from a Th17-dominated response to a Th1- and CD8^+^ T cell-dominated response (Hailemichael et al., 2022). Clinical trials are assessing the safety and tolerability of tocilizumab with PD-1 and CTLA-4 targeting drugs (NCT04940299).

TTI-101, the latest STAT3 inhibitor, has received orphan drug designation by the FDA for HCC treatment. It has been indicated that TTI-101 could inhibit neuropathic pain caused by cancer treatment (Kasembeli et al., 2021). Additionally, TTI-101 exhibited antitumor activity and clinical benefits in patients with solid tumors (NCT03195699). N4, another well-studied and novel STAT3 inhibitor, specifically binds to STAT3 and prevents the phosphorylation of tyrosine 705. N4 inhibits the proliferation and viability of pancreatic cancer cells in a dose-dependent manner and also inhibits migration and invasion *in vitro*. Importantly, in mouse models, N4 demonstrates a significant inhibitory effect on the growth and metastasis of pancreatic tumors. Additionally, N4 treatment inhibits TAM infiltration and the EMT process (Chen et al., 2021). However, N4 is still in the preclinical stage of development (Table 1).

**TABLE 1:** Therapeutic strategies targeting TAMs-mediated EMT process.

Target	Therapeutic agent	Mechanism	Therapeutic potential	Cancer types	References
M-CSF/CSF-1R	Pexidartinib (PLX3397)	Blocks CSF-1R tyrosine kinase activity to directly inhibit tumor growth, reduce TAM infiltration, and improve the immune microenvironment	Upregulates BAX to induce apoptosis in CSF1R-high cancer cells; inhibits ERK to reduce TAM infiltration and polarization; suppresses Treg cells to enhance CD8^+^ T cell infiltration	TGCT, Sarcoma, MPNST	(Thongchot et al., 2023), (Fujiwara et al., 2021), (Smeester et al., 2020), (Okojie et al., 2023), (Patwardhan et al., 2014)
M-CSF/CSF-1R	Emactuzumab (RG7155)	Binds to CSF-1R to reduce the recruitment and survival of TAMs, efficacy can be impacted by IL-4	Inhibits TAM infiltration and demonstrates synergy with other therapies	Advanced solid tumors	(Pradel et al., 2016b), (Gomez-Roca et al., 2022)
M-CSF/CSF-1R	BLZ945	Block CSF-1R tyrosine kinase activity to reduce TAM infiltration	Inhibits TAM and MDSC infiltration and enhances CD8^+^ T cell infiltration	HCC, OSCC, Advanced solid tumors	(He et al., 2021), (Guo et al., 2020), (Alcazar et al., 2024)
CCL2/CCR2	PF-04136309	Inhibits CCR2 signaling to reduce TAM infiltration and metastasis	Inhibits TAM and MDSC infiltration and enhances CD8^+^ T cell infiltration	PDAC, CRC	(Sanford et al., 2013), (Nywening et al., 2018), (Dang et al., 2021), (Noel et al., 2020b), (Nywening et al., 2016b)
CCL2/CCR2	BMS-813160	Inhibits CCR2/CCR5 signaling to reduce TAM infiltration and metastasis	Inhibits TAM and Treg infiltration and enhances CD8^+^ T cell infiltration, combined with chemotherapy or PD-(L)1 inhibitors	PDAC, NSCLC, HCC	(Venturini et al., 2023), (Le et al., 2018)
TGF-β	Galunisertib (LY2157299)	Inhibits TGFβR1 kinase activity	Directly inhibits the EMT process; Inhibit activation of TAMs and CAFs	PC, HCC, PDAC, RCC	(Holmgaard et al., 2018b), (Wu et al., 2022c), (Melisi et al., 2021b)
TGF-β	Fresolimumab	Neutralizes all three isoforms of TGF-β	Directly inhibits the EMT process; Improve immune microenvironment	Metastatic BC NSCLC, Melanoma	(Formenti et al., 2018), (Morris et al., 2014), (Arjaans et al., 2012)
IL-6	Tocilizumab	Binds to both soluble and membrane-bound IL-6 receptors to block IL-6 function	Directly inhibits the EMT process; Inhibits TAM polarization	HCC, BC	(Wan et al., 2014b), (Weng et al., 2019b)
IL-6/STAT3	TTI-101	Targets the STAT3 SH2 domain	Directly inhibits the EMT process	HCC	Tsimberidou et al. (2021)
IL-6/STAT3	N4	Targets the STAT3 SH2 domain	Directly prevents the phosphorylation of tyrosine 705	PDAC	Chen et al. (2021)

## 6 Controversies and gaps

While the role of TAMs in driving EMT is well-documented, the literature presents several conflicting findings that necessitate a more nuanced discussion. For instance, the dual role of TAMs in cancer progression and suppression remains a contentious area (Aras and Zaidi, 2017).

For instance, TAMs have been shown to enhance EMT and metastasis by secreting factors, which activate signaling pathways in tumor cells leading to increased invasiveness. This pro-tumorigenic role is evident in GC, where high levels of TAM infiltration correlate with increased EMT marker expression and poorer patient outcomes (Zhang et al., 2016). Conversely, research in CRC presents a different perspective. In a CRC model, TAMs were found to be pro-inflammatory and inhibited the proliferation of tumor cells. Additionally, these TAMs produced chemokines that attract T cells, stimulated the proliferation of allogeneic T cells, and activated type-1 T cells associated with anti-tumor immune responses (Ong et al., 2012).

Interestingly, the phenomenon cannot be solely attributed to the “pro-inflammatory” nature. Recent studies have shown that pro-inflammatory macrophages with high expression of IL-1β can promote the progression of PDAC (Caronni et al., 2023). Despite all being digestive tract tumors, the differences are still significant. Furthermore, numerous reports indicate that even M1 macrophages, typically associated with anti-tumor activity, can support tumor progression (You et al., 2022; Tsai et al., 2021). This dichotomy underscores the complexity of TAM behavior within the TME.

In addition, the differences in some research results are likely due to the choice of TAM model. Different experimental systems, including THP-1 derived macrophages, human peripheral blood mononuclear cells (PBMCs), murine BMDMs, and macrophages isolated directly from tumors, each have unique advantages and limitations that can affect study outcomes. THP-1 derived macrophages, originating from a human monocytic cell line, are popular due to their ease of use and consistency. However, they often fail to fully recapitulate the complexity and heterogeneity of primary TAMs. Human PBMCs, isolated from blood and differentiated into macrophages *in vitro*, offer a more physiologically relevant source. However, donor variability and the influence of *ex vivo* differentiation conditions can result in significant heterogeneity. Murine BMDMs are widely used in preclinical research due to their ease of obtaining large numbers of cells and genetic manipulation. Nonetheless, murine models may not always accurately reflect human macrophage biology. Macrophages isolated directly from tumors represent the most relevant model for studying TAM function within the TME. These cells have been exposed to the unique biochemical milieu of the tumor, including hypoxia, metabolic byproducts, and tumor-derived signals. However, the isolation process can be technically challenging and may alter macrophage phenotypes. Moreover, transitioning TAMs from a 3D tumor environment to 2D culture conditions can significantly impact their behavior. The current priority is to find the most suitable and universal TAM model for research. For example, a 2019 study provided a detailed comparison of different *in vitro* induction methods to obtain the most realistic TAM phenotypes (Benner et al., 2019).

## 7 Conclusion and future direction

TAMs, integral to the TME, play a pivotal role in cancer progression, influencing tumor growth, invasion, and metastasis. Their capacity to induce EMT stands as a key mechanism propelling cancer metastasis. This review has sought to collate and synthesize recent advancements in understanding the modalities through which TAMs orchestrate EMT and the potential therapeutic interventions aimed at curtailing TAM-mediated EMT.

**FIGURE 4:**
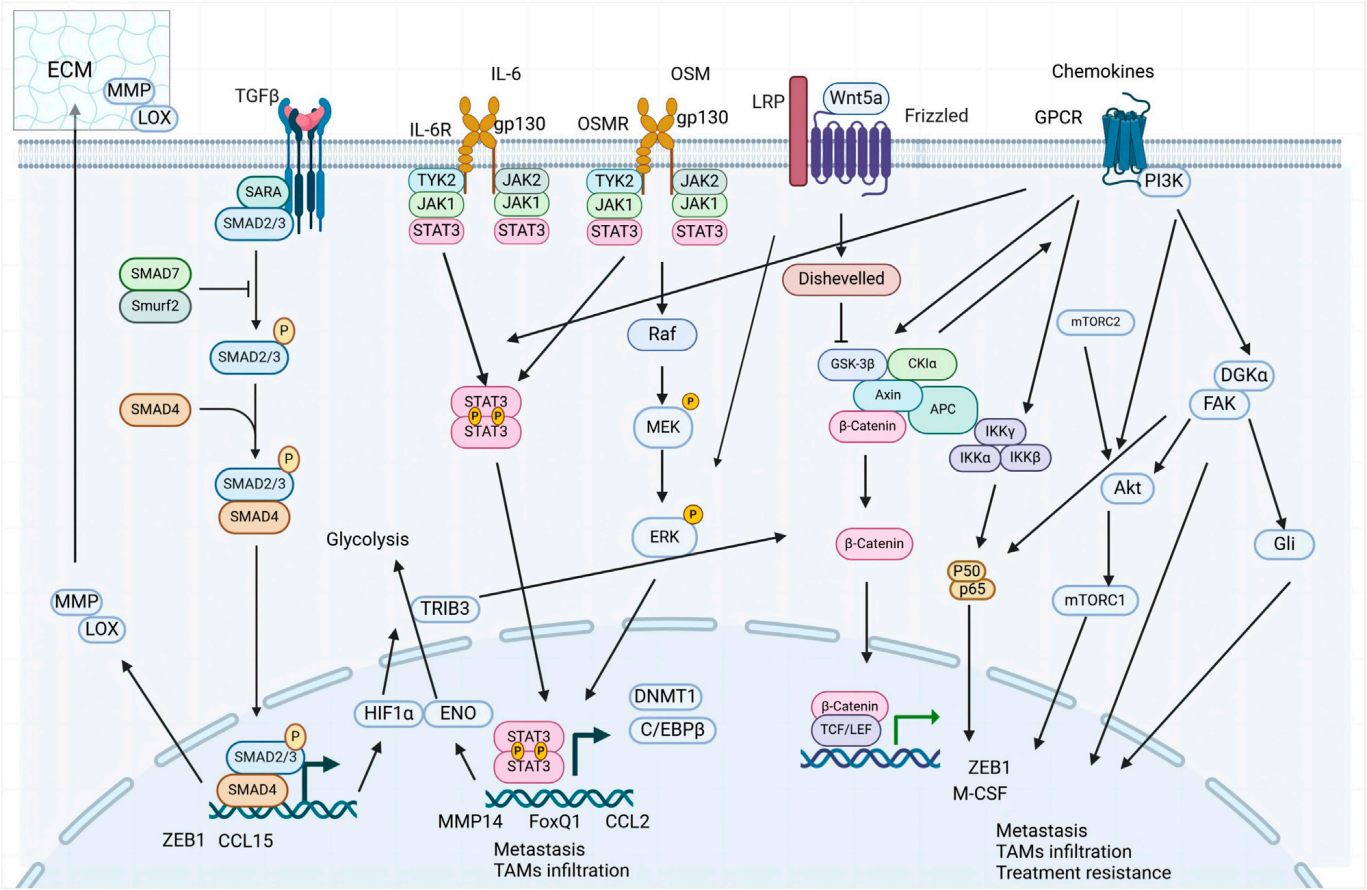
Key signaling pathways involved in tumor mesenchymal progression. This figure presents an overview of critical signaling pathways implicated in tumor mesenchymal progression, highlighting the complex network of interactions that contribute to various aspects of tumor biology, including metastasis, immune cell infiltration, and treatment resistance.

TAMs expedite the EMT process via the secretion of an array of cytokines, growth factors, and chemokines, culminating in the upregulation of EMT-inducing transcription factors within tumor cells. These transcription factors subsequently downregulate epithelial markers and upregulate mesenchymal markers, endowing cancer cells with enhanced invasive and metastatic capabilities. Furthermore, TAMs, as predominant immune cells within solid tumors, contribute significantly to the EMT process by fostering an inflammatory milieu, enabling tumor immune evasion, and facilitating interactions with other stromal cells. To elucidate the complex signaling pathways, the specific EMT signals occurring within tumors are detailed in Figure 4.

Various therapeutic approaches are being explored to disrupt TAM-induced EMT, including strategies aimed at reducing TAM recruitment, inhibiting TAM-secreted factors, and blocking the signaling pathways involved in TAM-driven EMT. Notably, interventions targeting the CSF/CSFR and CCL2/CCR2 signaling axes have shown promise in curtailing TAM recruitment, while inhibitors of key TAM-derived cytokines such as TGF-β and IL-6 are being investigated to hinder the pro-metastatic activities of TAMs. The ultimate goal of these strategies is to disrupt the interactions between TAMs and cancer cells, thereby preventing the EMT and acquisition of stemness traits in tumor cells.

However, it is crucial to acknowledge that TAMs are implicated in adverse clinical outcomes across a broader spectrum of cancers, including colorectal (Hou et al., 2024), ovarian (Schweer et al., 2022), and gastric cancers (Cao et al., 2023), among others. Due to space constraints, this review has focused on specific cancers such as breast cancer (including TNBC and IBC), lung cancer (particularly NSCLC), pancreatic cancer, and prostate cancer, which offer unique insights into TAM-mediated EMT and therapeutic interventions. Future research should aim to explore and summarize the role of TAMs in these additional cancer types, providing a more comprehensive understanding of their impact on cancer progression and therapeutic resistance.

Despite the promising developments in TAMs, several challenges persist in this field. The heterogeneity and plasticity of TAMs complicate efforts to selectively target them without affecting the normal functions of macrophages (Wang S. et al., 2024). Moreover, the intricate dynamics of TAM-tumor interactions and their variable impact on cancer progression across different contexts and cancer stages necessitate further investigation. The precise molecular signals and pathways driving the polarization of macrophages towards a pro-tumor phenotype in the TME remain inadequately understood (Vadevoo et al., 2021; Zhang A. et al., 2021). Recent studies have reported that lactate in the TME can promote the M2-like TAM phenotype, making lactylation in TAMs a promising direction for future research (Su et al., 2023). Additionally, the activation of β-catenin also facilitates the M2-like TAM phenotype (Tigue et al., 2023). These findings suggest potential targets for therapeutic intervention. Strategies to inhibit these pathways could help in reprogramming TAMs towards a more anti-tumor phenotype. However, these approaches need to be further explored and validated in clinical settings.

The role of TAMs in cancer metastasis requires further elucidation. Specifically, understanding how TAMs contribute to the metastatic spread of cancer cells and identifying the key factors involved in this process are critical areas of ongoing research. Developing effective strategies to therapeutically target TAMs without compromising normal immune functions remains a crucial objective. Furthermore, the interaction between TAMs and CSCs is another area requiring investigation. TAMs can support CSC maintenance and promote their stemness properties, which are associated with increased resistance to therapies and metastatic potential. Targeting this interaction could be a promising strategy to prevent cancer recurrence and metastasis. Lastly, identifying reliable biomarkers for monitoring TAM activity and therapeutic responses in cancer patients is essential. Biomarkers would enable the assessment of treatment efficacy and guide therapeutic decisions, ensuring more personalized and effective cancer treatment approaches.

As research in this area progresses, it is hoped that a deeper understanding of TAM-mediated mechanisms in cancer will lead to more effective and targeted therapeutic strategies, ultimately improving outcomes for patients with metastatic cancers.

## Figures and Tables

**Table:** 

**TAM**	tumor associated macrophage
**TME**	tumor microenvironment
**EMT**	epithelial-mesenchymal transition
**ECM**	extracellular matrix
**MMP**	matrix metalloproteinase
**CSF1R**	colony-stimulating factor 1 receptor
**RTM**	tissue-resident macrophage
**BMDM**	bone marrow-derived macrophage
**CCL**	C-C motif chemokine ligand
**M-CSF**	macrophage colony-stimulating factor
**GM-CSF**	granulocyte-macrophage colony-stimulating factor
**TNF-α**	tumor necrosis factor alpha
**IL-1β**	interleukin 1 beta
**IFN-γ**	interferon-gamma
**TGF-β**	transforming growth factor beta
**BC**	breast carcinoma
**LPS**	lipopolysaccharide
**STAT**	signal transducer and activator of transcription
**IRF**	interferon regulatory factor
**PPAR-γ**	peroxisome proliferator-activated receptors gamma
**VEGF**	vascular endothelial growth factor
**DNMT**	DNA methyltransferase
**ARG1**	arginase 1
**HDAC**	histone deacetylase
**OXPHOS**	oxidative phosphorylation
**FAO**	fatty acid oxidation
**NF-κB**	nuclear factor kappa B
**OSM**	oncostatin M
**EMT-TF, EMT**	associated transcription factor
**CRC**	colorectal cancer
**HIF-1α**	hypoxia-inducible factor 1 alpha
**TRIB3**	tribbles pseudokinase 3
**Wnt**	Wingless/Integrated
**Gli2**	glioma-associated homologue 2
**ER**	endoplasmic reticulum
**CSC**	cancer stem cell
**ESCC**	esophageal squamous cell carcinoma
**TNBC**	triple-negative breast cancer
**CAF**	cancer-associated fibroblast
**FGF**	fibroblast growth factor 2
**GC**	gastric cancer
**TGFBR3**	transforming growth factor beta receptor 3
**PDAC**	pancreatic ductal adenocarcinoma
**MSC**	mesenchymal stromal cell
**OSCC**	oral squamous cell carcinoma
**ENO1**	alpha-enolase
**THBS1**	thrombospondin 1
**Jak**	Janus kinase
**FoxQ1**	forkhead box Q1
**ZEB1**	zinc finger E-box binding homeobox 1
**PITPNM3**	phosphatidylinositol transfer proteinmembrane-associated 3
**SCCHN**	squamous cell carcinoma of the head and neck
**MTDH**	metadherin
**AnxA2**	Annexin A2
**PI3K**	phosphoinositide 3-kinase
**Akt**	protein kinase B
**GSK3β**	glycogen synthase kinase 3 beta
**GBC**	gallbladder cancer
**CaMKⅡ**	Calcium/Calmodulin-dependent protein kinase II
**ERK**	extracellular signal-regulated kinase
**FAK**	focal adhesion kinase
**DGKα**	diacylglycerol kinase alpha
**Treg**	regulatory T cell
**5-FU**	5-fluorouracil
**CXCL**	C-X-C motif chemokine ligand
**HGF**	hepatocyte growth factor
**HCC**	hepatocellular carcinoma
**PD-L1**	programmed death ligand 1
**MDSC**	myeloid-derived suppressor cell
**CM**	conditioned medium
**ROS**	reactive oxygen species
**EC**	endothelial cell
**GBM**	glioblastoma
**IBC**	inflammatory breast cancer
**YAP**	yes-associated protein
**OTUD5**	otu deubiquitinase 5
**GLUT3**	glucose transporter 3
**NSCLC**	non-small cell lung cancer
**EGFR**	epidermal growth factor receptor
**TKI**	tyrosine kinase inhibitor
**CB2**	cannabinoid receptor 2
**Oct4**	octamer-binding transcription factor 4
**Sox2**	SRY-box 2
**USP4**	ubiquitin-specific protease 4
**SUCNR1**	succinate receptor 1
**SV**	Simvastatin
**LOXL2**	lysyl oxidase-like protein 2
**PDX**	patient-derived xenograft
**AR**	androgen receptor
**ADT**	androgen deprivation therapy
**FN1**	fibronectin
**ITGA5**	integrin alpha 5
**SRC**	tyrosine kinase Src
**CTSK**	Cathepsin K
**CRPC**	castration-resistant prostate cancer
**SPDEF**	SAM-pointed domain-containing ETS transcription factor
**TGCT**	tenosynovial giant cell tumor
**irAE**	immune-related adverse event
**MCT-1**	monocarboxylate transporter 1
**ICB**	immune checkpoint blockade
